# The application of structural and machine learning models to predict the default risk of listed companies in the Iranian capital market

**DOI:** 10.1371/journal.pone.0292081

**Published:** 2023-11-27

**Authors:** Pejman Peykani, Mostafa Sargolzaei, Negin Sanadgol, Amir Takaloo, Hamidreza Kamyabfar

**Affiliations:** 1 School of Industrial Engineering, Iran University of Science and Technology, Tehran, Iran; 2 Department of Finance and Banking, Faculty of Management and Accounting, Allameh Tabataba’i University, Tehran, Iran; 3 School of Management, Economics and Progress Engineering, Iran University of Science and Technology, Tehran, Iran; Universiti Utara Malaysia, MALAYSIA

## Abstract

Inattention of economic policymakers to default risk and making inappropriate decisions related to this risk in the banking system and financial institutions can have many economic, political and social consequences. In this research, it has been tried to calculate the default risk of companies listed in the capital market of Iran. To achieve this goal, two structural models of Merton and Geske, two machine learning models of Random Forest and Gradient Boosted Decision Tree, as well as financial information of companies listed in the Iranian capital market during the years 2016 to 2021 have been used. Another goal of this research is to measure the predictive power of the four models presented in the calculation of default risk. The results obtained from the calculation of the default rate of the investigated companies show that 50 companies listed in the Iranian capital market (46 different companies) have defaulted during the 5-year research period and are subject to the Bankruptcy Article of the Iranian Trade Law. Also, the results obtained from the ROC curves for the predictive power of the presented models show that the structural models of Merton and Geske have almost equal power, but the predictive power of the Random Forest model is a little more than the Gradient Boosted Decision Tree model.

## 1. Introduction

Identifying types of risk, measuring them accurately and trying to minimize them is one of the most important goals of financial sciences worldwide [1–34]. One of the most important risks that has been in semi-traditional and modern human societies for a long time is default risk, or in more general terms, credit risk with default and bankruptcy [35–71]. The bankruptcy of financial institutions and various industries during the great credit crisis of 2008 is a sign of the importance of default risk and credit risk in the world economy [44, 47, 72–82]. In Iran, the unprofitability of some companies, which ultimately leads to their default and bankruptcy (being subject to Bankruptcy Article), has been one of the old problems of the financial markets.

Another problem that default risk creates for the financial markets, especially the debt market, is that in order to avoid the repetition of problems, strong guarantees are considered for the applicants for issuing debt securities. Finally, most of these guarantees cause problems for the government and government-owned banks [51]. These guarantees practically make the issuance of debt securities impossible or expensive for many companies. On the other hand, it creates obligations on the banking system that are not fundamentally related to it [83–86]. Credit rating is considered as an alternative method for guaranteeing securities; But this method also has its associated costs and is mostly reserved for large-sized companies. Increasing the ability of financial institutions, including banks, institutional investors such as investment companies, holding companies, and investment funds, in estimating credit risk is one of the necessities for the growth and maturity of the debt market. This issue causes these financial institutions to allocate loans to companies with a better view and face less risk of bankruptcy of debtors and related costs, including financial, economic and social costs [87–98].

It is very important to mention that one of the missing links of the default risk is to try to get a correct understanding and picture of the credit risk of each applicant for loans and debt securities. Obviously, with such a possibility, the lending institution can have a more accurate estimate of the applicant, and a more active primary and secondary market for debt securities will be created, in which debt securities are valued according to the risk and the investor invests in these securities according to the risk and expected return [99–104].

We know that many researches have been done on credit risk. In this research, an attempt has been made to examine default risk. Until now, research has been based on models where one of their simplifying assumptions is that the debt is coupon-free. In this research, Geske model is used, which has removed this assumption and the company can have coupon debt. Also, due to the increasing popularity of machine learning models, two machine learning techniques called Forest and Gradient Boosted Decision Tree have also been used to estimate the default risk of companies. Random Forest and Gradient Boosted Decision Tree are used for the first time to estimate the default risk of Iranian capital market companies. In this research, it has been tried to predict the default risk of companies in Iran’s capital market by using the four proposed models. Also, another achievement of this research will be to measure the predictive power of four default risk measurement models that can be used in financial institutions, including banks, financing companies, rating companies, and investment companies.

The rest of this article is organized as follows. In section 2, researches that have been done on this issue in the past are mentioned. In section 3, all the equations of the proposed models are stated, which were necessary to achieve the goal of this article. Also, in section 4, the results obtained from the proposed models are analyzed based on the information used. Finally, in section 5, the final conclusions are stated and the suggestions for the future to expand the concept are introduced.

## 2. Literature review

In this section, an overview of applied research in the field of default risk prediction is presented, which examines the default of different financial institutions using different models. We have tried to show the research done on structural models and models based on learning machine in Table 1.

**Table 1:** Conducted researches on structural models and machine learning.

**Structural Models**								
**Research**	**Description**							
Jones et al. [105]	Examining the Merton Model in the Pricing of Corporate Bonds							
Ogden [106]	Identifying the Constancy of the Interest Rate As the Main Problem of the Merton Model							
Zhou [107]	Disappointing Practical Application of Structural Models							
Layden & Saraniti [108]	Comparison of Merton and Schwartz Models							
Ho Eom et al. [109]	Review of Five Models of Merton, Gaske, Longstaff-Schwartz, Leland-Toft and Collin-Goldstein for Pricing 182 Corporate Bonds							
Jarrow et al. [110]	Providing a Robust Test of Merton Model for Credit Risk							
Schäfer et al. [111]	Investigating Credit Risk for An Asset Portfolio using the Jump-Diffusion Process							
Schaefer & Strebulaev [112]	Accurate Forecasting of the Sensitivity of Bond Returns to Changes in Equity Value (Coverage Ratios)							
Khansari & Fallahshams [113]	Predicting the Bankruptcy of Legal Clients of Iranian Banks using the KMV Model and Assessing the Accuracy of the Model							
Arora et al. [114]	Comparison of Credit Risk Structural Models of Merton, Vasicek-Kealhofer and Hull-White							
Liang & Jiang [115]	Modification of Black-Cox and Merton Structural Models using Indifference Pricing							
Gadzo et al. [116]	Investigating the Effect of Credit and Operational Risk on the Financial Performance of Global Banks Using PLS-SEM							
Pasricha et al. [117]	Proposing a Jump-Diffusion Model of Company Valuation using the Merton Model							
Our Research	Predicting the Default of Companies Listed in the Iranian Capital Market using Structural Models and Determining the Predictive Power of the Models for the First Time							
**Machine Learning Algorithms**								
**Research**	**Logistic Regression**	**Support Vector Machine**	**Neural Network**	**AdaBoost**	**Decision Tree**	**Random Forest**	**Gradient Boosted Decision Tree**	**Other**
Khandani et al. [118]						**✓**		
Brown & Mues [119]	**✓**	**✓**	**✓**		**✓**	**✓**		
Fitzpatrick & Mues [120]	**✓**					**✓**		**✓**
Panahi et al. [121]								**✓**
Chakraborty & Joseph [122]						**✓**		
Jones [123]								**✓**
Barboza et al. [124]	**✓**	**✓**	**✓**			**✓**		**✓**
Fallahpour et al. [125]		**✓**						
Fuster et al. [126]						**✓**		
Zhu et al. [127]	**✓**	**✓**			**✓**	**✓**		
Son et al. [128]							**✓**	
Deng et al. [129]							**✓**	
Sigrist et al. [130]							**✓**	
Acharya et al. [131]							**✓**	
Yıldırım et al. [132]	**✓**				**✓**	**✓**	**✓**	
Our Research						**✓**	**✓**	

As can be seen in Table 1, it is the first time that a combination of structural models and machine learning algorithms have been investigated using the financial information of companies in Iran’s capital market.

## 3. Methodology

In this section, we intend to express the equations used in the proposed models and the different conditions of the models. In this research, two classical structural models including Merton and Geske models and two models based on machine learning methods including models based on the Random Forest method and the Gradient Boosted Decision Tree method are used. In this article, we have tried to compare structural models and machine learning algorithms for the first time about the Iranian capital market. The aim of the research is to achieve the best estimate of the default risk of capital market companies in Iran, and to achieve this goal, the financial information of all capital market companies during the years 2016 to 2021 has been used.

In the first stage, we prepare a list of all capital market companies in the years 2016 to 2021. Then, among these companies, the companies that have been subject to Bankruptcy Article of the Iranian Trade Law during this period are identified. According to Bankruptcy Article of the Iranian Trade Law, if at least half of a company’s capital is lost, the board of directors must immediately invite the shareholders to an extraordinary general meeting to discuss the issue of liquidation or survival of the company.

### 3.1 Merton and Geske models

The Black-Scholes pricing equation is as follows:

$$s_{0}=VN\left(d_{1}\right)-Ke^{-rT}N\left(d_{2}\right)$$ (1)

In Eq 1, *s*_0_, *V*, *T* and *r* are respectively the value of the company’s shares, the value of the company’s assets, the maturity of the debt and the risk-free interest rate. In addition, in the Black-Scholes equation, *σ*_*v*_ means the price fluctuations of the Underlying Asset (the company’s assets), which is considered as the fluctuation of the company’s asset value in the Merton model.

In Geske model, it is assumed that the company has two types of long-term and short-term debt. The nominal value of the company’s short-term debt is called *M*_1_ and the nominal value of the company’s long-term debt is called *M*_2_. It is also assumed that the maturity of the company’s short-term debt is *T*_1_ and the maturity of the long-term debt is *T*_2_, with the assumption that *T*_2_ is greater than *T*_1_. Also, we assume that the company’s debts have a coupon in the middle of the year. At the time of coupon payment (short-term debt), if the value of the company’s assets is equal to the nominal value of the short-term debt and the value of the long-term debt, the shareholders will pay the debt, otherwise the company will default. As a result, we can consider the minimum value of the company to not default at the time of payment of short-term debt or $\bar{V}$, equal to Eq 2, where $B_{2T_{1}}$ is equal to the value of long-term debt at *T*_1_.


$$\bar{V}=M_{1}+B_{2T_{1}}$$ (2)

Eq 2 becomes Eq 3 considering that in any company, the value of debt is equal to assets minus equity.


$$\bar{V}=M_{1}+\bar{V}-M_{T_{1}}=M_{1}+\bar{V}-\bar{V}N\left(K_{2}+\sigma_{v}\sqrt{T_{2}-T_{1}}\right)+M_{2}e^{-r_{F_{1}}\left(T_{2}-T_{1}\right)}N\left(K_{2}\right)$$ (3)

According to the Geske model, the company’s stock value at time t is the following equation, and it is worth mentioning that *N*() function is a bivariate cumulative normal distribution function:

$$\begin{array}{l} S=VN_{2}\left(K_{1}+\sigma_{v}\sqrt{T_{1}-t}.K_{2}+\sigma_{v}\sqrt{T_{2}-t};\rho \right)-M_{2}e^{-r_{F_{2}}\left(T_{2}-t\right)}N_{2}\left(K_{1}.K_{2};\rho \right)\\ -M_{1}e^{-r_{F_{1}}\left(T_{2}-t\right)}N\left(K_{1}\right).\;\rho =\sqrt{\left(T_{1}-t)/(T_{2}-t\right)} \end{array}$$ (4)

Also, *K*_1_ and *K*_2_ are expressed in Eq 5.


$$K_{1}=\frac{ln\;\left(\frac{V}{\bar{V}}\right)+\left(r_{F_{1}}-\frac{1}{2}\sigma_{v}^{2}\right)\left(T_{1}-t\right)}{\sigma_{v}\sqrt{T_{1}-t}}.\;K_{2}=\frac{ln\left(V/M_{2}\right)+\left(r_{F_{2}}+\frac{1}{2}\sigma_{v}^{2}\right)\left(T_{2}-t\right)}{\sigma_{v}\sqrt{T_{2}-t}}$$ (5)

In Geske model, it is assumed that companies pay off their debt at the maturity of the coupon by issuing new shares for the benefit of creditors. The value of $\bar{V}$ is the amount that if the value of the company is lower at the time of coupon payment, the company will not be able to issue new shares. Also, the probability of default in *T*_1_ or *T*_2_, the probability of default in *T*_1_ and the future probability of default in *T*_2_ if there is no default in *T*_1_ are shown in Eqs 6 to 8, respectively.


$$RNDP_{T_{r}}=1-N_{2}\left(K_{1}.K_{2};\rho \right)$$ (6)

$$RNDP_{G_{S}}=1-N\left(K_{1}\right)$$ (7)

$$RNDP_{G_{F}}=1-\frac{N_{2}\left(K_{1}.K_{2};\rho \right)}{N\left(K_{1}\right)}$$ (8)

Finally, the required variables in the proposed models are presented in the Table 2.

**Table 2:** Variables used in structural models.

Parameter	Symbol	Merton Model	Geske Model
Company Stock Price (Market Value of Equity)	*s*_0_	✓	✓
Book Value of Debt	*K*	✓	
Interest Rate	*r*	✓	✓
Debt Maturity	*T*	✓	
Asset Value	*V*	✓	✓
Asset Volatility	*σ*_*v*_	✓	✓
Book Value of Short-term Debt (Coupon)	*M*_1_		✓
Maturity of Short-term Debt (Coupon)	*T*_1_		✓
Book Value of Long-term Debt	*M*_2_		✓
Maturity of Long-term Debt	*T*_2_		✓
Minimum Asset Price At the Time of Coupon Payment	$\bar{V}$		✓

In the following, the method of estimating the variables of the models is explained. The information related to the stock price of the companies as a determinant of the equity value has been extracted through Tseclient software. The book value of companies’ debts is obtained through Codal site and the risk-free interest rate is obtained through the Central Bank of Iran site. Also, the maturity of the debt is equal to 1 year. In Geske model, short-term debt and long-term debt are equal to the company’s current liabilities and non-current liabilities. The maturity date of short-term debt is 6 months and long-term debt is 1 year. It is worth mentioning that to determine $\bar{V}$, *σ*_*v*_ and *V*, by adding the famous equation of the relationship between the underlying asset price and the option price, it is done as follows:

$$\sigma_{s}=\frac{\partial S}{\partial V}\frac{V}{S}\sigma_{v}$$ (9)

By adding Eq 9 and based on a numerical algorithm, and solving the proposed equations, the unknown parameters are obtained. To calculate the parameters, we first give an initial value to V and *σ*_*v*_. Then we set the initial value of the asset value equal to the sum of the market value of the company’s stock and the nominal value of its debt, and calculate the initial value of the fluctuation of the asset value by setting ∂S/∂V and the fluctuation value of the company’s stock price equal to 1. Then, using the obtained values, we calculate *d*_1_ and *d*_2_, and the model provides values of *S* and *σ*_*s*_. Considering the real values of *S* and *σ*_*s*_ and the values obtained from the model, it is tried to minimize Eq 10 by changing the value of *V* and *σ*_*v*_.


$${\left(\frac{S_{t_{Model}}}{S_{Observed}}-1\right)}^{2}+{\left(\frac{\sigma_{s_{Model}}}{\sigma_{s_{Observed}}}-1\right)}^{2}$$ (10)

The above equation for Merton’s model can be solved through Excel software and using the solver plugin. For Geske model, it is done from Python software and Scipy library and by solving two Eqs 4 and 9.

### 3.2 Machine learning models

In models based on machine learning algorithms, data needs to be labeled. First, a list of companies that are subject to Bankruptcy Article in the years 2016 to 2021 is prepared and labeled as defaulter companies. In Table 3, the symbol of the companies that subject to Bankruptcy Article in the years 2016 to 2021 is presented. In total, the data of 44 different companies subject to Bankruptcy Article has been presented, and then according to the mentioned procedure, the information of one year of healthy companies is also examined. It should be noted that the data set is divided into two parts based on the priority of the financial years and the training and test data sets are created. As a result, the data of the financial years leading to 2016 to 2019 were used as training data and the information of the companies in the financial years leading to 2020 and 2021 were also used as the test data set, which includes 9 companies subject to Bankruptcy Article and 91 healthy companies. The rest of the information, including the information of 206 companies, is used as a training dataset. It is important to mention that no changes are applied to the data and the data is used in raw form. In the model based on the Random Forest method, first the dataset is divided into a large number of random subsets. Then a tree is drawn for each subset. It should be noted that the explanatory variables of each tree are also selected based on a number of variables randomly selected from among all variables. Finally, each data is selected using the majority of labels given to it by different trees [124, 132].

**Table 3:** Companies subject to Bankruptcy Article during 2016 to 2021.

2016	2017	2018	2019	2020	2021
ST001	ST051	ST207	ST001	ST005	ST002
ST002	ST052	ST208	ST012	ST059	ST057
ST003	ST053	ST103	ST160	ST207	ST239
ST004	ST054	ST104	ST161	ST208	ST241
ST005	ST055	ST105	ST162		ST242
ST006	ST056	ST106	ST163		
ST007	ST057	ST107	ST175		
ST008	ST058	ST108			
ST009	ST059				
ST010	ST060				
ST011	ST061				
ST012	ST062				
ST013					
ST014					

The data related to the information of the test data set companies are used to estimate the default of these companies. In the model based on the Gradient Boosted Decision Tree model and unlike the Random Forest model which is based on multiple decision trees, a tree is used to classify assets. In this model, an effort is made to focus on the error of the tree and by using this way, after each classification stage, more weight is given to the samples whose classification was wrong, and the error is reduced. Continue this method until the error reaches zero and the basic method of Gradient Boosted Decision Tree is used.

In the model based on Random Forest and Gradient-Boosted Decision Trees, the information of defaulter companies is used in each year along with number of variables of that company. The used variables can be a wide range of variables based on the registered and legal information of the company, such as the type of company, shareholders, date and place of registration, subject of activity, etc., to variables based on financial statements, such as types of financial ratios and gross and operating profit margins. or include variables based on the company’s activity and operations, such as the type of product or service produced or provided, or information related to the company’s stock price.

Since our field of study is companies whose shares are traded on the capital market, it has been tried to use variables in two categories, variables based on financial statements and variables based on information related to the company’s stock price, as the most basic information available to the company in the capital market.

According to the aforementioned, the variables of the training and test data sets are displayed in Table 4 [124].

**Table 4:** Variables used in machine learning algorithms.

Variable	Description
X1	Net Working Capital to Total Assets
X2	Accumulated Interest on Total Assets
X3	Income Before Taxes and Interest on Total Assets
X4	Market Value to Debt Value
X5	Sales to Total Assets
OM	Profit Before Tax and Interest on Sales
GA	Asset Growth Rate
GS	Sales Growth Rate
GE	Growth Rate of the Number of Employees
CROE	ROE Growth
CPB	P/B Growth

In this research, the ROC parameter is used to check the predictive power of the probabilities provided by each model and the parametric t and Wilcoxon tests to measure the existence of differences between the models. By using four indices of confusion matrix, several indices are calculated to estimate the strength of the model from different dimensions. How to calculate some of these indicators is shown in Table 5.

**Table 5:** Indexes for measuring classification models.

Index	Calculation Method	Description
Sensitivity-TPR	$TPR=\frac{TP}{TP+FN}$	1 − Type II Error
Specificity-TNR	$TNR=\frac{TN}{TN+FP}$	1 − Type I Error
Precision	$Precision=\frac{TP}{TP+FP}$	Measuring the Accuracy of the Positive Values Provided by the Model
Accuracy (ACC)	$ACC=\frac{TP+TN}{TP+TN+FP+FN}$	Overall Accuracy Measurement Index of the Model

True Positive (TP) is data that is correctly detected as positive. For example, in a default detection model of a company, if the healthiness of the company is considered positive, a company that is healthy in the real world will be correctly classified by the healthy model. TP is known as the correct identification of the model. Also, False Positive (FP) is data that is mistakenly considered positive. In the previous example it is equal to a defaulter company that is mistakenly recognized as a healthy company. FP is equivalent to Type I Error in the statistical hypothesis test. Next, it should be mentioned that True Negative (TN) is data that is correctly recognized as negative, such as a defaulter company whose default is correctly predicted. Finally, False Negative (FN) is data that is wrongly detected as negative, meaning a healthy company that is wrongly classified as a defaulter. FN is equivalent to Type II Error in statistical hypothesis test.

ROC is a parameter by which the ability of the model is measured using TPR and FPR indices. The TPR index or model sensitivity measure calculates the ratio of detected TPs by the model to all positive samples. The FPR index also calculates the model error rate using the ratio of detected FPs to total negative samples. In continuous classification models, a threshold type is defined on the continuous output as a criterion for the final classification of the data. For example, in the default risk measurement model of the investigated companies, the output for each company is obtained in the form of a probability of default. Considering a probability as a threshold, companies are divided into two healthy or defaulter categories. By changing the threshold value, different values of TPR and FPR are obtained. If the threshold value is considered high, defaulter firms will be less likely to be classified as healthy, which means the FPR will be low. On the other hand, it is possible that a number of healthy companies will be classified as defaulters, which will reduce the TPR. If the threshold value is considered low, it is more likely that the defaulter companies will be identified as healthy companies and the FPR value will be higher. The ROC curve is drawn in a two-dimensional space where the vertical column is TPR and the horizontal column is FPR, based on different thresholds. Finally, for each threshold, there will be a different value of TPR and FDR. The most favorable point for a model will be the point (1, 0) in the northwest of the figure. The line drawn in the figure and passing through the points (0, 0), (0.5, 0.5) and (1, 1) shows the performance of a random classification model. It is worth noting that the model with a larger surface area under the ROC figure is a more desirable model. The surface area under the figure is a number between 0 and 1.

In order to compare each pair of models in this research, the data generated by the models are analyzed as ordered pairs (each pair consisting of the possibilities of a company). Also, the T- Statistic is obtained based on Eqs 11 and 12.


$$t=\frac{{\bar{X}}_{A}-{\bar{X}}_{B}}{\sqrt{\frac{S^{2}}{n_{A}}+\frac{S^{2}}{n_{B}}}}$$ (11)

$$S^{2}=\;\frac{\sum_{i=1}^{n_{A}}{\left(X_{i}-{\bar{X}}_{A}\right)}^{2}+\sum_{i=1}^{n_{B}}{\left(X_{i}-{\bar{X}}_{B}\right)}^{2}}{n_{A}+n_{B}-2}$$ (12)

In the above equations, the variables *X*_*A*_, *X*_*B*_, *n*_*A*_, *n*_*B*_ and *S*^2^ are respectively the sample of the first model, the sample of the second model, the number of samples of the first model, the number of samples of the second model and the mixed variance of the data of both models. In this test, the hypothesis H0 is equal to the equality of the average of the two samples and the hypothesis H1 is equal to the rejection of the hypothesis H_0 and as a result the inequality of the average of the two samples. Like the T-test, the Wilcoxon test is designed to compare the average of two populations, with the difference that in this test, the assumption of normality of the statistical population is not important.

For Wilcoxon test for n data by each model (2n in total), we need to go through 5 steps. In the first step, the absolute value of the difference of both pairs of data is |*X*_*Ai*_ − *X*_*Bi*_| is calculated. Then, for the difference of both pairs, the value of the sgn function is obtained, which is defined as 1 if the difference is positive (*X*_*Ai*_ > *X*_*Bi*_) and -1 if it is negative, and 0 if it is 0. In the second step, zero is removed from the values obtained from the sgn function. In the third step, the values obtained from Absolute function are arranged in increasing order. In the fourth step, the sorted Absolute values are ranked, with the lowest rank being given 1. If the two Absolute function values are equal, the average ranks are assigned to them. The rank assigned to the output of the Absolute function of each pair (i) is denoted as *R*_*i*_. Finally, w statistic is determined according to Eq 13.


$$w=\sum_{i=1}^{n_{r}}\left[sgn\left(x_{2.i}-x_{1.i}\right).R_{i}\right]$$ (13)

The distribution of w statistic is equal to zero and its variance is obtained from Eq 14.


$$\sigma_{w}^{2}=\frac{n_{r}\left(n_{r}+1\right)\left(2n_{r}+1\right)}{24}$$ (14)

In the Wilcoxon test, the statistical assumptions are the same as the T-test, and finally, according to the obtained value of w and comparing it with the critical value, the hypothesis H0 is confirmed or rejected.

## 4. Results

In this section, findings resulting from the implementation of structural models and machine learning algorithms are presented. Also, the point that should be mentioned here is that the complete results obtained from the models examined in this research are displayed in the Appendices A to D in S1 File.

Merton model has been implemented using Excel software, while Geske model and models based on Random Forest and Gradient Boosted Decision Tree have been analyzed using Python software. First, a table of the frequency of listed companies subject to Bankruptcy Article based on different industries in Iran’s capital market is presented in Table 6.

**Table 6:** Companies subject to Bankruptcy Article by industry.

Industry	Number of Companies	Industry	Number of Companies
Automobile	5	Cement	2
Iron and Steel	1	Non-Metallic Minerals	5
Metal Products	1	Sugar	2
Metal Ore	1	Machinery	2
Precious Metals	1	Coal	1
Chemical	6	Household Appliances	1
Real Estate	1	Ceramic Tiles	1
Fertilizer and Nitrogen Compounds	1	Transportation	2
Agriculture	3	Pulp and Paper	1
Dairy Products	2	Rubber and Plastic	1
Auto parts	3	Telecommunication Equipment	2
Medicine	1	Textile	1
Engineering Activities	2	Wood	1

From the point of view of the number of companies included in each year, it seems that this trend has decreased during the years 2016 to 2021, which is evident in Table 7.

**Table 7:** Number of companies subject to Bankruptcy Article in 2016 to 2021.

Year	2016	2017	2018	2019	2020	2021
**Number of Companies**	14	12	8	7	4	5

Now, the statistical indicators of each feature including average, standard deviation, minimum, first quartile, second quartile, third quartile and maximum are presented in three formats of all companies, defaulted companies and healthy companies respectively in Tables 8–10.

**Table 8:** Statistical indicators of data set characteristics—All companies.

Indicator	X1	X2	X3	X4	X5	OM	GA	GS	GE	CROE	CPB
**Average**	0.1200	0.1351	0.1809	7.3667	0.9389	-0.021	0.5703	0.4641	0.0885	1.5227	1.3981
**Standard Deviation**	0.3285	0.3132	0.1747	18.5045	0.7458	4.9992	3.9764	1.1517	0.7734	27.9210	10.4087
**Minimum**	-2.704	-2.896	-0.385	0.0939	0.0000	-86.70	-0.5710	-0.8906	-0.550	-40.8270	-33.946
**First Quartile**	-0.033	0.0163	0.0531	1.2994	0.5024	0.0682	0.0402	0.0286	-0.038	-0.2791	-0.1950
**Second Quartile**	0.1525	0.1376	0.1447	2.6478	0.7537	0.1806	0.1815	0.2794	0.0000	0.0101	0.1183
**Third Quartile**	0.2945	0.2855	0.2808	6.5478	1.1788	0.3584	0.3981	0.5999	0.0472	0.2738	0.9407
**Maximum**	0.7923	0.8240	0.7405	262.2955	5.1235	7.9113	68.6112	13.4856	9.6156	481.4055	137.07

**Table 9:** Statistical indicators of data set characteristics–Defaulted companies.

Indicator	X1	X2	X3	X4	X5	OM	GA	GS	GE	CROE	CPB
**Average**	0.1644	0.1919	0.2071	8.2750	0.9854	0.2652	0.3452	0.4573	0.0690	1.8457	0.4253
**Standard Deviation**	0.2741	0.2474	0.1696	20.0365	0.7297	0.2761	0.6861	0.9081	0.5879	30.5035	2.1258
**Minimum**	-2.185	-2.301	-0.097	0.0939	0.0360	-0.3656	-0.5710	-0.5955	-0.534	-40.8270	12.4545
**First Quartile**	0.0238	0.0714	0.0800	1.4772	0.5545	0.0975	0.0478	0.0469	-0.027	-0.2505	-0.2016
**Second Quartile**	0.1760	0.1873	0.1763	2.8084	0.8150	0.2034	0.1837	0.3000	0.0022	0.0195	0.0897
**Third Quartile**	0.3137	0.3131	0.3028	7.6517	1.2073	0.3797	0.4145	0.6220	0.0521	0.3059	0.6631
**Maximum**	0.7923	0.8240	0.7405	262.2955	5.1235	2.9979	8.8094	10.5012	8.9584	481.4055	16.3036

**Table 10:** Statistical indicators of data set characteristics—Healthy companies.

Indicator	X1	X2	X3	X4	X5	OM	GA	GS	GE	CROE	CPB
**Average**	-0.112	-0.158	0.0440	2.7349	0.7023	-1.527	1.7517	0.5111	0.1924	-0.127	6.518
**Standard Deviation**	0.4697	0.4384	0.1352	4.0073	0.7967	12.4757	9.8119	2.0095	1.3968	2.6659	25.154
**Minimum**	-2.704	-2.896	-0.385	0.2003	0.0000	-86.707	-0.2007	-0.8906	-0.550	-13.80	-33.94
**First Quartile**	-0.231	-0.249	-0.011	0.7359	0.3007	-0.0387	0.0126	-0.1060	-0.102	-0.387	0.0428
**Second Quartile**	-0.042	-0.082	0.0333	1.2733	0.4603	0.0476	0.1291	0.1082	-0.037	-0.002	1.6420
**Third Quartile**	0.1392	-0.013	0.0853	2.7338	0.8481	0.1081	0.3193	0.4829	0.0440	0.1313	5.2090
**Maximum**	0.5951	0.6362	0.4854	20.5936	4.8914	7.9113	68.6112	13.4856	9.6156	10.2780	137.07

**Fig 1:**
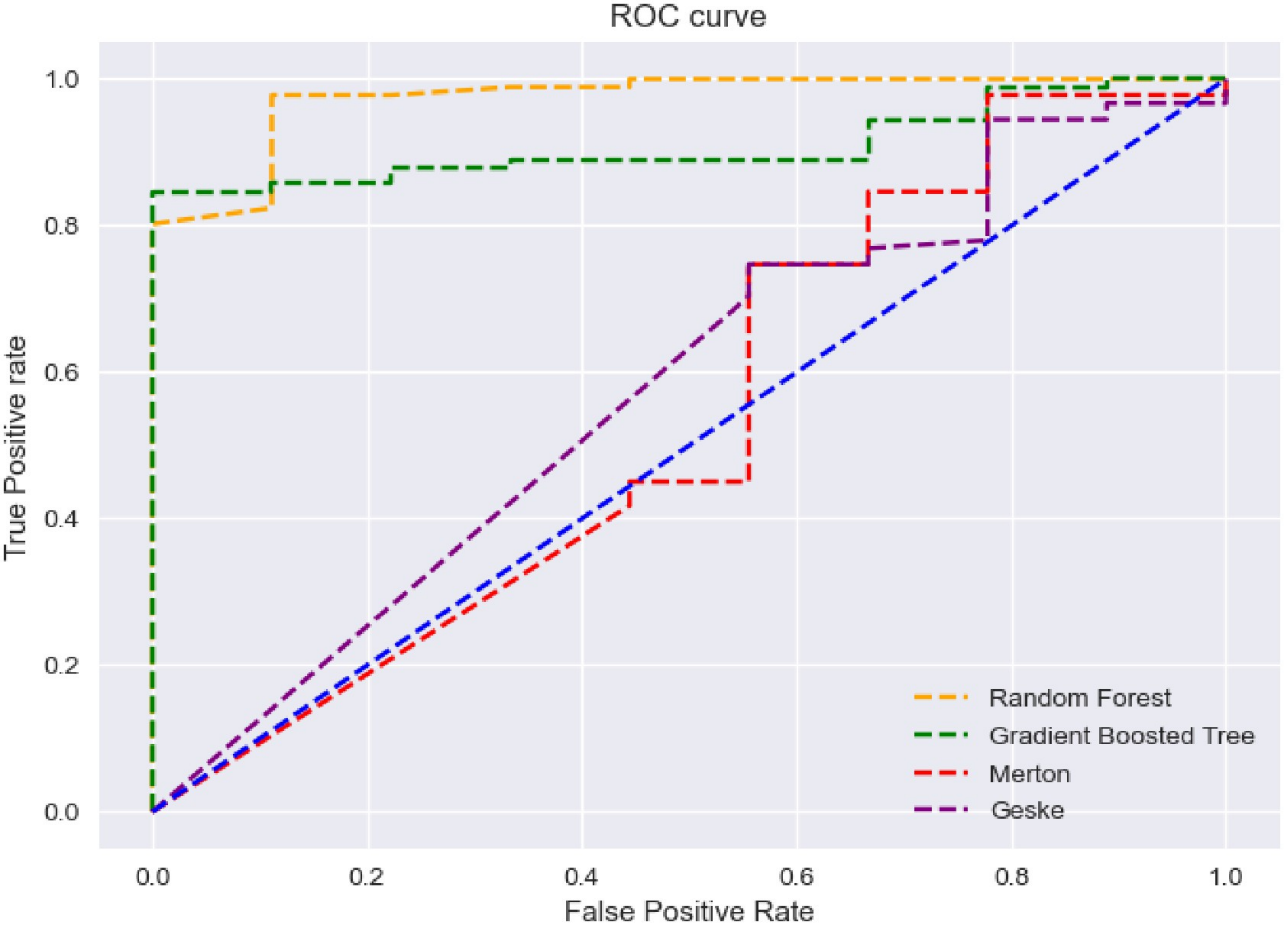
ROC curves for four models.

After implementing the models in different situations, we have investigated the capabilities of each model using the ROC. The entire dataset used includes 306 companies (50 companies subject to Bankruptcy Article and 256 healthy companies). It should be noted that the information of 206 companies, including 40 companies subject to Bankruptcy Article and 166 healthy companies, was used as training data between 2016 and 2019. Also, 100 companies, including 9 companies subject to Bankruptcy Article and 91 healthy companies between 2020 and 2021, have been used as test data. Machine learning algorithms have been implemented on all data sets through the use of training data and test data. Then the structural models have been implemented on the data of 2020 and 2021 and finally the performance of the used models have been compared using ROC. In Fig 1, the Roc curve for all four models is displayed. A model with a higher area under the curve has better performance. Considering that the number of the surface area under the ROC curve is between 0 and 1, if this number is closer to 1, the model has more power.

According to the numerical results presented in Table 11, the model based on the Random Forest method with the ROC curve area of 0.97 has the highest power to detect defaulted companies. Then, the model based on the Gradient Boosted Decision Tree method, with the area under the ROC curve of 0.91, ranks next. The numbers related to structural models have a significant gap with models based on machine learning techniques, so that the score of Geske model is 0.58 and Merton model is recorded with a lower number of 0.53, and they are in the next ranks from the point of view of recognition power. It should also be noted that the models have been compared two by two using the Wilcoxon model and the T-test, and the results of the statistical tests are displayed on the left side of the results table.

**Table 11:** Research results.

Model	ROC-AUC	Comparison of Models					
		Model	RF	GBDT	Geske	Merton	
**RF**	0.97	**RF**	-	0.51932921	4.85e-13	2.46e-12	T-test’s P-Value
**GBDT**	0.91	**GBDT**	0.00074705	-	1.91e-6	5.34e-6	
**Geske**	0.58	**Geske**	2.89e-17	3.16e-17	-	0.2893528	
**Merton**	0.53	**Merton**	1.87e-16	4.93e-12	7.73e-14	-	
			Wilcoxon’s P-Value				

Among the performance comparison tests of the models, only the similarity of the performance of the two models based on Random Forest and Gradient Boosted Decision Tree has been confirmed based on the t-test statistic. Models based on Random Forest and Gradient Boosted Decision Tree have closer performance. In Table 12, some indicators of measuring the power of two models are presented.

**Table 12:** Comparative indices of machine learning models.

Model	TN	FN	FP	TP	Accuracy	Sensitivity	Specificity	Precision
**RF**	7	2	2	89	96%	98%	78%	98%
**GBDT**	7	2	11	80	87%	98%	39%	88%

Among the indicators presented in Table 12 and the AUC number, the AUC number is the most important. After the AUC number, a parameter that is important in financial helplessness forecasting models (default and bankruptcy), is the specificity of the model, which measures the model’s ability to predict unhealthy companies. The model based on Gradient Boosted Decision Tree has given poor performance on unbalanced data set. In addition, the sensitivity of the model also measures its accuracy among healthy companies. The Accuracy index also measures the overall accuracy of the model results, although both models have shown good performance, but the performance of the Random Forest model is clearly better. Finally, the Precision index measures the ability of the model to identify healthy companies in the data set. Due to the importance of data features in machine learning algorithms, models have been re-implemented in different cases by removing one or more features.

**Fig 2:**
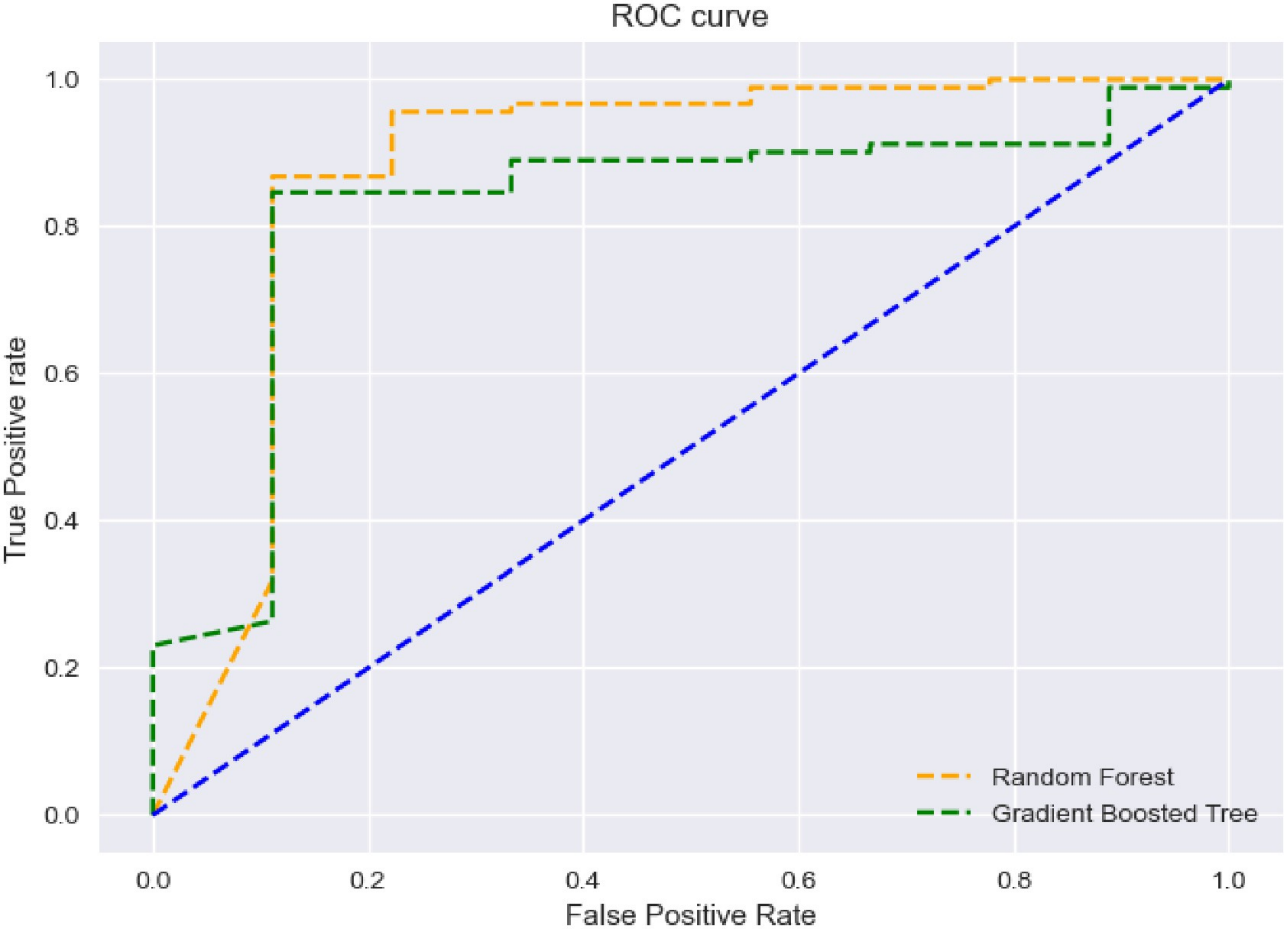
ROC curves for machine learning based on models with Altman features.

The ROC curve in Fig 2 is obtained using the features used in the Altman model (X1 to X5). In this case, the surface area under Fig 2 is equal to 0.88 and 0.83 for each of the models based on Random Forest and Gradient Boosted Decision Tree. As a result, it can be claimed that the research results of Barboza et al. [124] have been repeated in this research.

In order to measure the importance of each feature, we remove one feature each time and implement the models. The ROC number after removing each feature individually is presented in Table 13.

**Table 13:** ROC number rfter removing each feature.

Removed Feature	RF	GBDT
X1	98	90
X2	97	97
X3	97	90
X4	97	90
X5	97	91
OM	97	90
GA	97	92
GS	97	92
GE	97	90
CROE	98	90
CPB	94	80

As can be seen, based on the data in Table 13, the CPB feature, which is the change in the company’s P/B ratio, has a great impact on the explanatory power of machine learning algorithms. In another case, structural models have been implemented on all data consisting of company information from 2016 to 2021.

**Fig 3:**
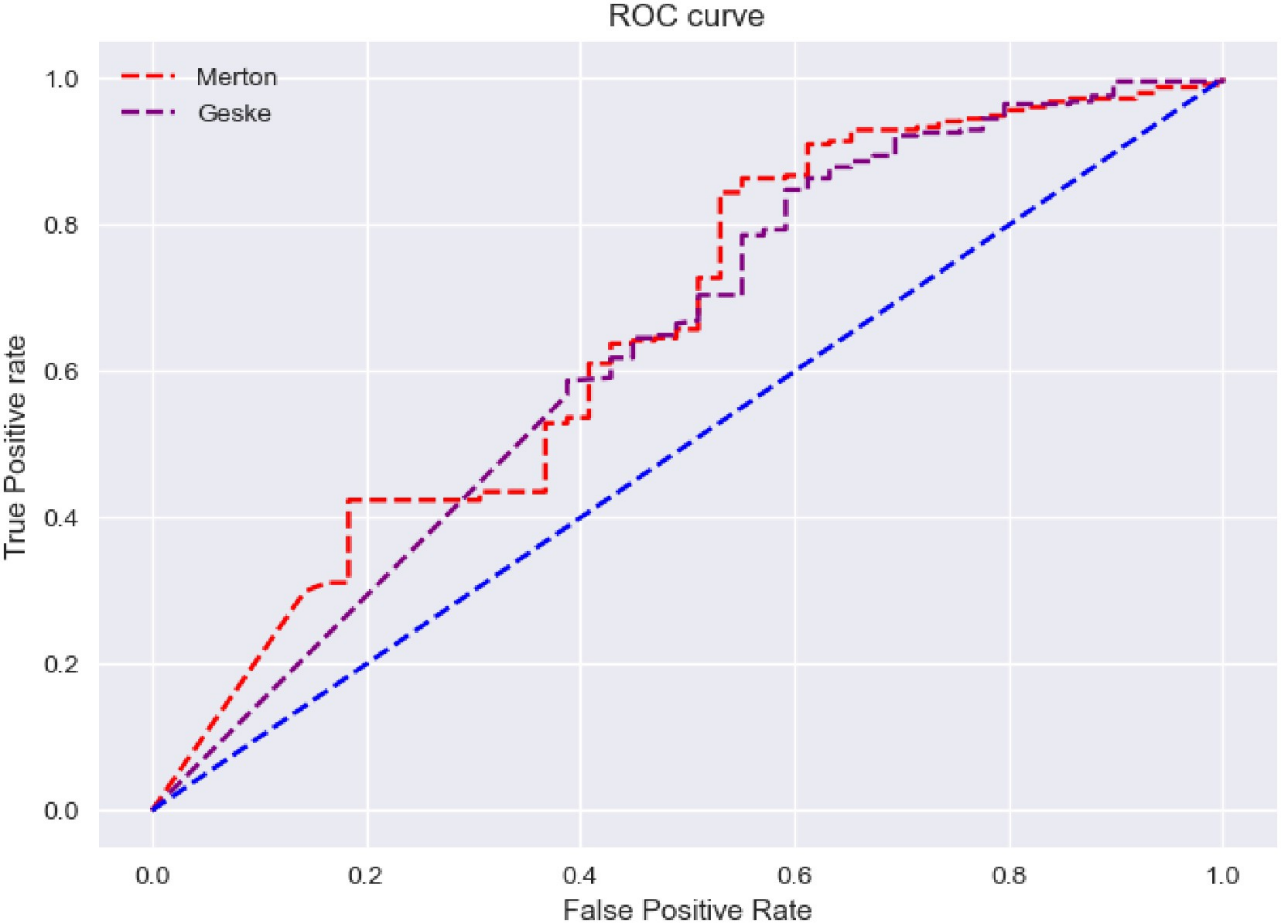
ROC curves in implementation mode on data from 2016 to 2021.

**Fig 4:**
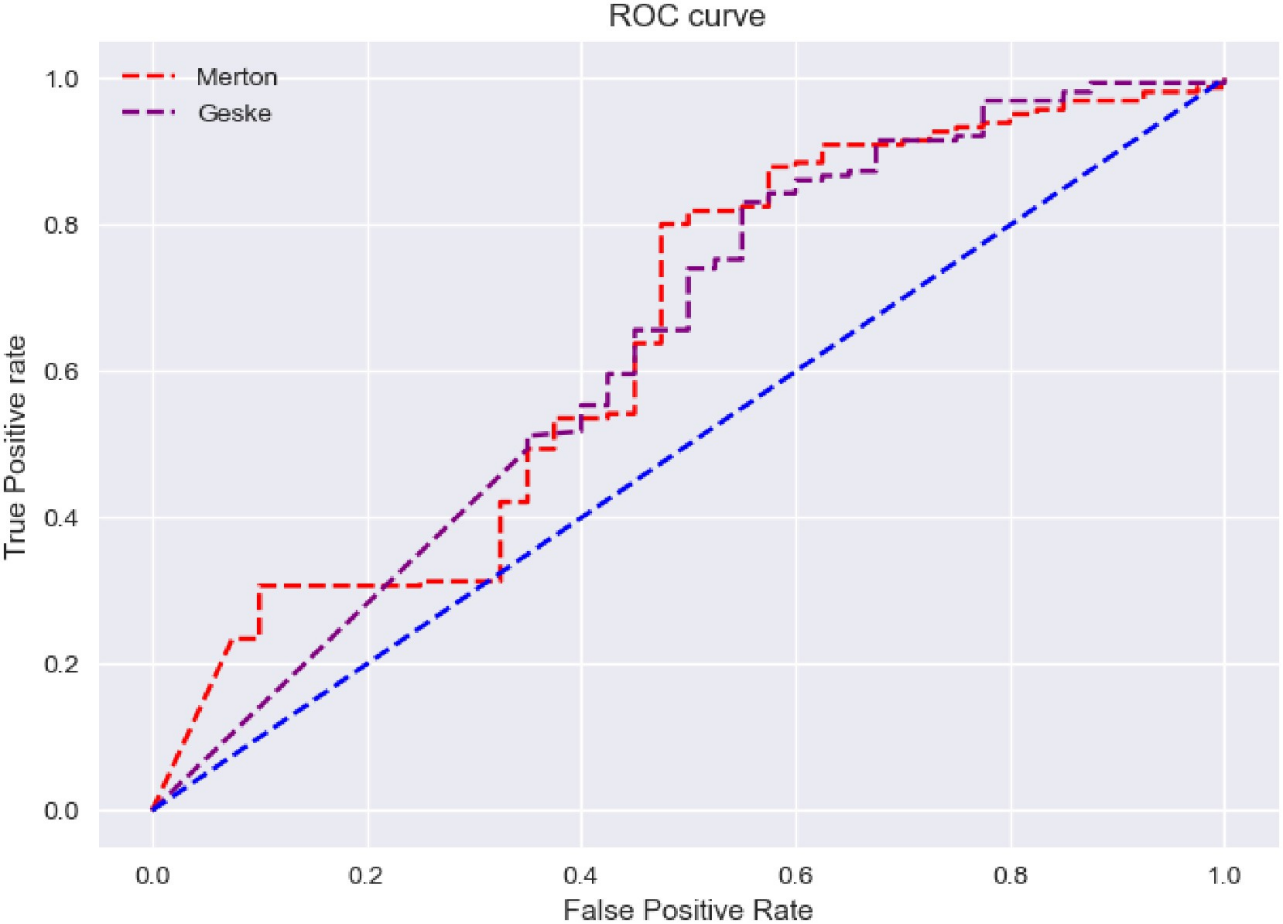
Implementation of structural models on data from 2016 to 2019.

As can be seen in Fig 3, with the increase of the used data, the area under the ROC curve of Merton and Geske models improves and reaches 0.65 and 0.63. Considering the general increase in stock prices as a result of the historical growth of the Iranian capital market in the first half of 2020, and the reliance of structural models on stock prices and price fluctuations, these models have been used for the data from 2016 to 2019, and their performance results are shown in Fig 4.

The obtained numbers as the area under Fig 4, similar to the previous case, were 0.65 and 0.63, which seems that the market developments in 2019 and 2020 have reduced the effectiveness of structural models.

Now we want to implement structural models on a different statistical sample. Due to not using data from 2016 to 2019 for structural models in the previous part, in this part, structural models for data from 2016 to 2021 are also used and the results are shown in Table 14.

**Table 14:** Prediction results of structural models for the entire data set.

Data	ROC-AUC Merton Model	ROC-AUC Geske Model	T-Statistic	Statistical Assumption	Wilcoxon Test Statistic	Statistical Assumption
2016 to 2021	0.65	0.63	4.75e-8	H0 is rejected	1.86e-30	H0 is rejected

As it is clear from the results of the ROC curve, the overall performance of the structural models improves with the increase in the number of data. By increasing the sample size, the performance of Merton model becomes better than Geske model, which is confirmed by the Wilcoxon and T-tests.

## 5. Conclusions and future research directions

Default risk measurement and its role in credit risk measurement is an important role in measuring the more general concept of risk in companies in the capital market. On the other hand, the credit assessment of the loan applicant has always been the most important concern of the credit supply side, including banks, financial institutions, investment funds, suppliers of raw materials and other investors. Various models have been introduced to measure the default risk of companies, and in this research, four models, including the Merton model, the Geske model, and models based on random forest and Gradient Boosted Decision Tree, have been evaluated, and the results of each model and the parameters for measuring the predictive power of each mode is provided. In this research, we have checked the capacity of each model using the ROC curve. The total dataset used includes 306 companies (49 companies subject to Bankruptcy Article and 256 healthy companies). It should be noted that the information of 206 companies including 40 companies subject to Bankruptcy Article and 166 healthy companies were used as training data between 2016 and 2019, and also 100 companies including 9 companies subject to Bankruptcy Article and 91 healthy companies between the years 2020 to 2021 have been used as test data. Machine learning algorithms are implemented on all datasets using training data and test data. Then the structural models have been implemented on the data of 2020 and 2021 and finally the performance of the used models has been compared using the ROC curve. According to the results obtained from the surface area under the ROC figure, it can be concluded that the Merton model and the Geske model have performed better than random models with ROC scores of 0.54 to 0.65 and 0.58 to 0.63 respectively for different researches. Also, according to the conditions governing this research and according to the results obtained from the surface area under the ROC figure, it is evident that the Random Forest and Gradient Boosted Decision Tree models are very powerful models for predicting the default risk of companies, respectively, with a ROC score of 0.98 and 0.91 and accuracy score 0.96 and 0.87. Finally, for future studies, data envelopment analysis (DEA) method [133–169] and machine learning algorithms can be combined to performance prediction of listed companies in the Iranian capital market. Also, DEA as a powerful performance measurement tool can be applied for assessing credit risk management of companies [170–186].

## Supporting information

S1 File (DOCX)
