# Supplementary material for: The application of structural and machine learning models to predict the default risk of listed companies in the Iranian capital market
Source: PLoS One. 2023 Nov 27;18(11):e0292081. doi: 10.1371/journal.pone.0292081 (PMC10681326; doi:10.1371/journal.pone.0292081)
Supplement: S1 File — (DOCX) [file pone.0292081.s001.docx]

**Appendix A**

**Table 15.** Merton Model Results

| **Company Name** | **Estimated Volatility of Asset Value** | **Estimated Asset Value** | **Probability of Default** | **Company Name** | **Estimated Volatility of Asset Value** | **Estimated Asset Value** | **Probability of Default** |
| --- | --- | --- | --- | --- | --- | --- | --- |
| **ST001** | %14.59 | 21760426$ | %21.74 | **ST154** | %15.46 | 2297644$ | %2.21 |
| **ST002** | %21.82 | 8581646$ | %4.44 | **ST155** | %6.37 | 8507528$ | %0.00 |
| **ST003** | %20.48 | 1430906$ | %1.43 | **ST156** | %5.37 | 62419436$ | %0.00 |
| **ST004** | %14.05 | 103637150$ | %0.03 | **ST157** | %28.71 | 4149616$ | %0.01 |
| **ST005** | %24.85 | 5786304$ | %12.54 | **ST158** | %15.46 | 37998782$ | %13.14 |
| **ST006** | %39.27 | 3633618$ | %0.28 | **ST159** | %43.38 | 36391758$ | %27.15 |
| **ST007** | %88.07 | 3385008$ | %53.46 | **ST160** | %92.80 | 6339618$ | %46.01 |
| **ST008** | %30.53 | 4169796$ | %1.72 | **ST161** | %55.15 | 2162722$ | %3.64 |
| **ST009** | %23.55 | 2031302$ | %6.18 | **ST162** | %32.81 | 9010746$ | %0.05 |
| **ST010** | %0.53 | 3276102$ | %0.00 | **ST163** | %29.22 | 13010030$ | %1.68 |
| **ST011** | %12.28 | 5092166$ | %8.08 | **ST164** | %61.84 | 16087656$ | %10.79 |
| **ST012** | %40.21 | 6870170$ | %20.69 | **ST165** | %24.13 | 6190516$ | %0.40 |
| **ST013** | %0.14 | 254598$ | %0.00 | **ST166** | %29.82 | 614237890$ | %0.00 |
| **ST014** | %40.49 | 901028$ | %0.00 | **ST167** | %19.50 | 20470504$ | %8.12 |
| **ST015** | %29.72 | 3861516$ | %0.63 | **ST168** | %31.80 | 2014268$ | %0.03 |
| **ST016** | %27.20 | 128422$ | %8.07 | **ST169** | %31.83 | 72659108$ | %0.05 |
| **ST017** | %11.45 | 502580894$ | %0.00 | **ST170** | %24.54 | 57104714$ | %0.00 |
| **ST018** | %16.67 | 20260780$ | %0.00 | **ST171** | %10.73 | 969808$ | %0.00 |
| **ST019** | %26.12 | 7528234$ | %2.62 | **ST172** | %26.92 | 6519264$ | %0.35 |
| **ST020** | %16.87 | 4859998$ | %0.47 | **ST173** | %35.58 | 5571280$ | %0.03 |
| **ST021** | %6.76 | 989754$ | %50.51 | **ST174** | %27.28 | 5717448$ | %1.27 |
| **ST022** | %32.88 | 257604$ | %0.01 | **ST175** | %36.00 | 5111134$ | %0.00 |
| **ST023** | %26.10 | 35946368$ | %5.51 | **ST176** | %14.50 | 26618114$ | %16.54 |
| **ST024** | %41.27 | 1412438$ | %0.00 | **ST177** | %22.06 | 33443040$ | %0.18 |
| **ST025** | %25.51 | 8986284$ | %9.99 | **ST178** | %24.44 | 9139708$ | %0.00 |
| **ST026** | %36.89 | 3809166$ | %0.00 | **ST179** | %44.90 | 4602532$ | %0.00 |
| **ST027** | %16.49 | 14728290$ | %0.51 | **ST180** | %36.26 | 5046850$ | %0.88 |
| **ST028** | %27.19 | 2883332$ | %0.05 | **ST181** | %18.95 | 6301392$ | %0.98 |
| **ST029** | %22.95 | 15996182$ | %0.02 | **ST182** | %25.42 | 1236625292$ | %0.00 |
| **ST030** | %7.49 | 7891710$ | %0.00 | **ST183** | %23.48 | 9857934$ | %0.00 |
| **ST031** | %18.60 | 148192918$ | %1.52 | **ST184** | %30.57 | 8010544$ | %0.02 |
| **ST032** | %14.38 | 5069090$ | %0.00 | **ST185** | %20.94 | 2403978$ | %1.45 |
| **ST033** | %17.74 | 927606$ | %0.06 | **ST186** | %19.11 | 57912826$ | %4.35 |
| **ST034** | %27.71 | 4376050$ | %0.00 | **ST187** | %27.23 | 59537442$ | %0.00 |
| **ST035** | %24.09 | 5784722$ | %0.06 | **ST188** | %29.33 | 1609508$ | %0.00 |
| **ST036** | %22.29 | 1904916$ | %0.05 | **ST189** | %54.06 | 5994316$ | %0.002 |
| **ST037** | %20.54 | 22315426$ | %0.00 | **ST190** | %22.63 | 6780314$ | %10.64 |
| **ST038** | %27.76 | 49194050$ | %30.99 | **ST191** | %22.25 | 12182924$ | %0.53 |
| **ST039** | %5.34 | 326728$ | %0.00 | **ST192** | %32.23 | 7301482$ | %0.00 |
| **ST040** | %35.31 | 993476$ | %0.02 | **ST193** | %26.39 | 684289214$ | %0.00 |
| **ST041** | %18.66 | 85687184$ | %0.00 | **ST194** | %14.35 | 4937268$ | %0.01 |
| **ST042** | %2.47 | 7170012$ | %0.00 | **ST195** | %35.53 | 2484032$ | %0.00 |
| **ST043** | %5.89 | 2114052$ | %0.00 | **ST196** | %26.52 | 4548304$ | %0.02 |
| **ST044** | %33.72 | 1313028$ | %0.70 | **ST197** | %26.91 | 89749142$ | %0.02 |
| **ST045** | %7.05 | 1698204$ | %0.00 | **ST198** | %27.18 | 2721478$ | %0.46 |
| **ST046** | %10.61 | 5610676$ | %0.00 | **ST199** | %29.53 | 3996914$ | %0.01 |
| **ST047** | %23.67 | 5181280$ | %0.00 | **ST200** | %31.30 | 6342724$ | %0.53 |
| **ST048** | %12.56 | 452814$ | %0.14 | **ST201** | %35.17 | 1356058$ | %0.13 |
| **ST049** | %18.89 | 9978662$ | %0.72 | **ST202** | %9.76 | 6888366$ | %0.00 |
| **ST050** | %21.47 | 5970964$ | %4.52 | **ST203** | %33.81 | 1027834$ | %0.44 |
| **ST051** | %29.74 | 673166$ | %0.00 | **ST204** | %36.35 | 1871012$ | %0.25 |
| **ST052** | %28.64 | 1631160$ | %3.71 | **ST205** | %34.98 | 3451864$ | %0.03 |
| **ST053** | %0.94 | 5441682$ | %0.00 | **ST206** | %34.93 | 3239688$ | %0.03 |
| **ST054** | %18.50 | 821026$ | %0.00 | **ST207** | %22.22 | 910705890$ | %19.78 |
| **ST055** | %20.54 | 1911176$ | %0.61 | **ST208** | %21.11 | 1154663476$ | %14.27 |
| **ST056** | %9.03 | 30022776$ | %0.00 | **ST209** | %30.54 | 19662770$ | %0.00 |
| **ST057** | %9.17 | 1226494$ | %2.11 | **ST210** | %21.68 | 19312364$ | %0.00 |
| **ST058** | %12.46 | 1176790$ | %43.21 | **ST211** | %35.33 | 6069416$ | %0.53 |
| **ST059** | %19.80 | 6631912$ | %8.29 | **ST212** | %27.13 | 1612932$ | %1.28 |
| **ST060** | %25.32 | 854624$ | %0.00 | **ST213** | %26.71 | 18644410$ | %23.51 |
| **ST061** | %22.20 | 2055522$ | %0.00 | **ST214** | %30.47 | 6286758$ | %0.12 |
| **ST062** | %12.44 | 2679052$ | %0.00 | **ST215** | %27.13 | 6445532$ | %12.81 |
| **ST063** | %2.09 | 15638740$ | %0.00 | **ST216** | %45.90 | 34968334$ | %0.00 |
| **ST064** | %20.27 | 1598320$ | %1.83 | **ST217** | %21.01 | 20464058$ | %22.04 |
| **ST065** | %18.28 | 70926782$ | %0.00 | **ST218** | %36.80 | 150876546$ | %0.05 |
| **ST066** | %26.82 | 4857820$ | %2.34 | **ST219** | %40.07 | 31385566$ | %0.00 |
| **ST067** | %13.98 | 3315320$ | %2.94 | **ST220** | %42.77 | 19614846$ | %0.00 |
| **ST068** | %28.05 | 3091634$ | %0.00 | **ST221** | %47.52 | 21735458$ | %0.00 |
| **ST069** | %17.93 | 5364088$ | %0.20 | **ST222** | %44.10 | 44567158$ | %0.12 |
| **ST070** | %21.35 | 4229534$ | %0.00 | **ST223** | %31.96 | 60264398$ | %0.00 |
| **ST071** | %14.89 | 4289096$ | %2.60 | **ST224** | %34.01 | 492866492$ | %0.02 |
| **ST072** | %10.99 | 104460522$ | %0.00 | **ST225** | %47.28 | 18206978$ | %0.00 |
| **ST073** | %16.74 | 3942346$ | %0.29 | **ST226** | %27.99 | 1156717018$ | %0.00 |
| **ST074** | %18.24 | 3166900$ | %0.95 | **ST227** | %44.68 | 21456986$ | %0.00 |
| **ST075** | %14.62 | 10048522$ | %0.00 | **ST228** | %28.16 | 66356378$ | %0.00 |
| **ST076** | %3.90 | 15885010$ | %0.00 | **ST229** | %36.13 | 83820252$ | %0.00 |
| **ST077** | %13.36 | 17159532$ | %0.00 | **ST230** | %31.88 | 860112658$ | %0.00 |
| **ST078** | %15.50 | 11383890$ | %0.00 | **ST231** | %28.80 | 624391084$ | %0.00 |
| **ST079** | %11.13 | 21792620$ | %7.39 | **ST232** | %45.89 | 26659714$ | %0.00 |
| **ST080** | %18.96 | 1615578$ | %8.31 | **ST233** | %41.59 | 16339154$ | %0.00 |
| **ST081** | %5.72 | 920610$ | %0.00 | **ST234** | %50.16 | 21954782$ | %0.00 |
| **ST082** | %30.38 | 6531022$ | %0.00 | **ST235** | %40.16 | 31355436$ | %0.29 |
| **ST083** | %34.97 | 17382582$ | %0.00 | **ST236** | %43.45 | 36811560$ | %0.00 |
| **ST084** | %22.97 | 10562690$ | %0.00 | **ST237** | %56.25 | 88798688$ | %0.00 |
| **ST085** | %27.37 | 1447518$ | %0.36 | **ST238** | %30.08 | 91060944$ | %0.05 |
| **ST086** | %12.07 | 129321966$ | %0.00 | **ST239** | %48.19 | 89805570$ | %0.00 |
| **ST087** | %26.79 | 2777292$ | %0.12 | **ST240** | %27.01 | 9658932$ | %0.00 |
| **ST088** | %24.13 | 47604478$ | %0.00 | **ST241** | %0.07 | 2376088$ | %0.00 |
| **ST089** | %5.57 | 20853422$ | %54.39 | **ST242** | %40.94 | 11674160$ | %0.00 |
| **ST090** | %20.10 | 52826284$ | %1.47 | **ST243** | %29.29 | 19588026$ | %0.00 |
| **ST091** | %22.74 | 1784974$ | %0.01 | **ST244** | %44.64 | 49843464$ | %0.01 |
| **ST092** | %23.60 | 2755778$ | %0.70 | **ST245** | %45.52 | 31826662$ | %0.00 |
| **ST093** | %24.32 | 3828948$ | %0.66 | **ST246** | %34.44 | 214241598$ | %0.00 |
| **ST094** | %11.07 | 193014$ | %0.00 | **ST247** | %31.31 | 48581600$ | %0.00 |
| **ST095** | %21.87 | 2641358$ | %0.57 | **ST248** | %40.00 | 19798264$ | %0.00 |
| **ST096** | %26.80 | 4220338$ | %0.42 | **ST249** | %24.62 | 21417720$ | %0.00 |
| **ST097** | %22.94 | 2268170$ | %0.05 | **ST250** | %26.28 | 15229874$ | %0.00 |
| **ST098** | %0.22 | 1931302$ | %0.00 | **ST251** | %38.66 | 123294806$ | %0.01 |
| **ST099** | %35.00 | 1679992$ | %0.00 | **ST252** | %46.79 | 83265474$ | %0.00 |
| **ST100** | %18.31 | 1858764$ | %0.38 | **ST253** | %45.37 | 31821346$ | %0.00 |
| **ST101** | %10.26 | 467014382$ | %6.34 | **ST254** | %26.91 | 178364590$ | %0.14 |
| **ST102** | %8.38 | 504200224$ | %6.93 | **ST255** | %43.96 | 51157630$ | %0.00 |
| **ST103** | %12.46 | 116797270$ | %6.22 | **ST256** | %57.94 | 44513306$ | %0.00 |
| **ST104** | %6.32 | 46814950$ | %0.00 | **ST257** | %39.33 | 167455064$ | %0.00 |
| **ST105** | %27.16 | 14550886$ | %9.77 | **ST258** | %32.25 | 96411524$ | %2.18 |
| **ST106** | %17.86 | 611434$ | %0.02 | **ST259** | %33.21 | 27063546$ | %0.00 |
| **ST107** | %45.88 | 2193252$ | %0.00 | **ST260** | %40.95 | 21165558$ | %0.00 |
| **ST108** | %25.31 | 1893042$ | %0.05 | **ST261** | %35.09 | 20951768$ | %0.00 |
| **ST109** | %48.12 | 1342984$ | %1.28 | **ST262** | %41.49 | 24972066$ | %0.00 |
| **ST110** | %25.33 | 3677756$ | %0.00 | **ST263** | %41.33 | 392970528$ | %0.00 |
| **ST111** | %17.10 | 4901174$ | %0.37 | **ST264** | %37.90 | 181364208$ | %0.01 |
| **ST112** | %12.76 | 1250560$ | %5.39 | **ST265** | %49.24 | 77372142$ | %0.00 |
| **ST113** | %10.61 | 84823614$ | %0.16 | **ST266** | %35.43 | 330298462$ | %0.00 |
| **ST114** | %17.29 | 45548620$ | %0.00 | **ST267** | %44.77 | 144077434$ | %0.00 |
| **ST115** | %22.03 | 1058030$ | %0.00 | **ST268** | %36.35 | 63993394$ | %0.00 |
| **ST116** | %19.26 | 78164438$ | %0.00 | **ST269** | %34.09 | 11300372$ | %0.00 |
| **ST117** | %16.54 | 2700714$ | %0.14 | **ST270** | %30.59 | 37374584$ | %1.52 |
| **ST118** | %18.83 | 34823064$ | %0.00 | **ST271** | %33.65 | 19485924$ | %0.00 |
| **ST119** | %9.19 | 226102816$ | %0.00 | **ST272** | %77.65 | 22721956$ | %0.09 |
| **ST120** | %26.25 | 1619844$ | %0.07 | **ST273** | %22.23 | 51020508$ | %0.00 |
| **ST121** | %26.42 | 6353308$ | %2.77 | **ST274** | %43.90 | 891244768$ | %0.01 |
| **ST122** | %27.25 | 3223832$ | %5.44 | **ST275** | %40.00 | 538171232$ | %0.00 |
| **ST123** | %16.12 | 5677036$ | %0.00 | **ST276** | %32.93 | 29080556$ | %0.00 |
| **ST124** | %22.85 | 3206834$ | %3.21 | **ST277** | %36.05 | 35705512$ | %1.56 |
| **ST125** | %32.37 | 1044732$ | %0.00 | **ST278** | %30.05 | 57720112$ | %0.00 |
| **ST126** | %177.41 | 1138452$ | %53.61 | **ST279** | %30.12 | 23901022$ | %0.00 |
| **ST127** | %23.83 | 4079608$ | %0.00 | **ST280** | %21.13 | 46669354$ | %0.01 |
| **ST128** | %17.92 | 7020600$ | %33.18 | **ST281** | %19.92 | 54142042$ | %0.00 |
| **ST129** | %25.27 | 5533556$ | %0.39 | **ST282** | %41.06 | 1208925950$ | %0.05 |
| **ST130** | %20.47 | 179932232$ | %0.00 | **ST283** | %7.60 | 4164028$ | %0.00 |
| **ST131** | %10.83 | 13028494$ | %0.02 | **ST284** | %44.75 | 58352276$ | %0.00 |
| **ST132** | %37.01 | 5567082$ | %3.10 | **ST285** | %49.63 | 165759082$ | %0.00 |
| **ST133** | %24.55 | 435332$ | %0.00 | **ST286** | %43.07 | 17982452$ | %0.00 |
| **ST134** | %7.20 | 23305178$ | %0.00 | **ST287** | %31.84 | 551139486$ | %0.00 |
| **ST135** | %60.67 | 3712180$ | %6.56 | **ST288** | %64.57 | 19352966$ | %0.00 |
| **ST136** | %17.19 | 33184520$ | %0.03 | **ST289** | %50.84 | 678373468$ | %0.00 |
| **ST137** | %20.06 | 887242$ | %0.06 | **ST290** | %50.24 | 1523831562$ | %0.00 |
| **ST138** | %22.23 | 80919172$ | %0.00 | **ST291** | %45.34 | 1081115592$ | %0.00 |
| **ST139** | %20.95 | 2141856$ | %0.00 | **ST292** | %40.41 | 43819736$ | %0.00 |
| **ST140** | %22.75 | 3174972$ | %0.00 | **ST293** | %38.19 | 26389162$ | %0.00 |
| **ST141** | %23.32 | 6397844$ | %0.00 | **ST294** | %26.06 | 3027988$ | %0.00 |
| **ST142** | %5.54 | 8764078$ | %0.00 | **ST295** | %89.66 | 24298620$ | %2.67 |
| **ST143** | %27.86 | 2382946$ | %0.00 | **ST296** | %45.89 | 76936464$ | %0.00 |
| **ST144** | %28.44 | 3321584$ | %0.00 | **ST297** | %4.41 | 42910094$ | %0.00 |
| **ST145** | %4.54 | 467597932$ | %0.00 | **ST298** | %45.01 | 55030762$ | %0.00 |
| **ST146** | %21.14 | 36983202$ | %0.00 | **ST299** | %40.21 | 72476952$ | %0.00 |
| **ST147** | %19.98 | 26708440$ | %0.29 | **ST300** | %39.32 | 39317460$ | %0.00 |
| **ST148** | %17.94 | 3820250$ | %2.89 | **ST301** | %21.59 | 11977748$ | %0.00 |
| **ST149** | %18.24 | 248003142$ | %0.00 | **ST302** | %39.74 | 13482012$ | %0.00 |
| **ST150** | %17.13 | 2559010$ | %11.92 | **ST303** | %44.64 | 44523724$ | %0.00 |
| **ST151** | %32.10 | 5930700$ | %0.29 | **ST304** | %43.90 | 487275928$ | %0.00 |
| **ST152** | %12.11 | 10498204$ | %0.27 | **ST305** | %85.96 | 21415214$ | %0.00 |
| **ST153** | %35.05 | 4489292$ | %0.00 | **ST306** | %51.03 | 40362778$ | %0.00 |

**Appendix B**

**Table 16.** Geske Model Results

| **Company Name** | **Estimated Volatility of Asset Value** | **Total Probability of Default** | **Total Probability of Short-term Default** | **Total Probability of Long-term Default** |
| --- | --- | --- | --- | --- |
| **ST001** | %11.67 | %0.03 | %0.00 | %0.03 |
| **ST002** | %19.44 | %0.00 | %0.00 | %0.00 |
| **ST003** | %19.51 | %0.00 | %0.00 | %0.00 |
| **ST004** | %13.63 | %0.00 | %0.00 | %0.00 |
| **ST005** | %22.71 | %0.15 | %0.00 | %0.15 |
| **ST006** | %38.53 | %0.00 | %0.00 | %0.00 |
| **ST007** | %65.74 | %27.11 | %5.38 | %22.97 |
| **ST008** | %29.59 | %0.01 | %0.00 | %0.01 |
| **ST009** | %22.03 | %0.02 | %0.00 | %0.02 |
| **ST010** | %0.50 | %0.00 | %0.00 | %0.00 |
| **ST011** | %11.05 | %0.00 | %0.00 | %0.00 |
| **ST012** | %34.89 | %2.65 | %0.17 | %2.49 |
| **ST013** | %0.13 | %0.00 | %0.00 | %0.00 |
| **ST014** | %40.04 | %0.00 | %0.00 | %0.00 |
| **ST015** | %28.75 | %0.00 | %0.00 | %0.00 |
| **ST016** | %25.02 | %0.10 | %0.00 | %0.10 |
| **ST017** | %11.21 | %0.00 | %0.00 | %0.00 |
| **ST018** | %16.09 | %0.00 | %0.00 | %0.00 |
| **ST019** | %24.92 | %0.01 | %0.00 | %0.01 |
| **ST020** | %20.42 | %0.00 | %0.00 | %0.00 |
| **ST021** | %4.40 | %0.15 | %0.00 | %0.14 |
| **ST022** | %32.29 | %0.00 | %0.00 | %0.00 |
| **ST023** | %24.65 | %0.03 | %0.00 | %0.03 |
| **ST024** | %41.02 | %0.00 | %0.00 | %0.00 |
| **ST025** | %23.16 | %0.11 | %0.00 | %0.11 |
| **ST026** | %36.43 | %0.00 | %0.00 | %0.00 |
| **ST027** | %15.84 | %0.00 | %0.00 | %0.00 |
| **ST028** | %26.58 | %0.00 | %0.00 | %0.00 |
| **ST029** | %22.27 | %0.00 | %0.00 | %0.00 |
| **ST030** | %7.37 | %0.00 | %0.00 | %0.00 |
| **ST031** | %17.66 | %0.00 | %0.00 | %0.00 |
| **ST032** | %14.04 | %0.00 | %0.00 | %0.00 |
| **ST033** | %17.11 | %0.00 | %0.00 | %0.00 |
| **ST034** | %27.48 | %0.00 | %0.00 | %0.00 |
| **ST035** | %23.36 | %0.00 | %0.00 | %0.00 |
| **ST036** | %21.61 | %0.00 | %0.00 | %0.00 |
| **ST037** | %20.44 | %0.00 | %0.00 | %0.00 |
| **ST038** | %21.43 | %1.86 | %0.10 | %1.76 |
| **ST039** | %5.12 | %0.00 | %0.00 | %0.00 |
| **ST040** | %34.61 | %0.00 | %0.00 | %0.00 |
| **ST041** | %18.18 | %0.00 | %0.00 | %0.00 |
| **ST042** | %2.40 | %0.00 | %0.00 | %0.00 |
| **ST043** | %5.71 | %0.00 | %0.00 | %0.00 |
| **ST044** | %32.77 | %0.00 | %0.00 | %0.00 |
| **ST045** | %6.89 | %0.00 | %0.00 | %0.00 |
| **ST046** | %10.24 | %0.00 | %0.00 | %0.00 |
| **ST047** | %23.38 | %0.00 | %0.00 | %0.00 |
| **ST048** | %12.01 | %0.00 | %0.00 | %0.00 |
| **ST049** | %18.05 | %0.00 | %0.00 | %0.00 |
| **ST050** | %20.57 | %0.00 | %0.00 | %0.00 |
| **ST051** | %29.28 | %0.00 | %0.00 | %0.00 |
| **ST052** | %27.33 | %0.02 | %0.00 | %0.02 |
| **ST053** | %0.89 | %0.00 | %0.00 | %0.00 |
| **ST054** | %18.02 | %0.00 | %0.00 | %0.00 |
| **ST055** | %19.72 | %0.00 | %0.00 | %0.00 |
| **ST056** | %8.77 | %0.00 | %0.00 | %0.00 |
| **ST057** | %8.51 | %0.00 | %0.00 | %0.00 |
| **ST058** | %7.60 | %0.16 | %0.00 | %0.15 |
| **ST059** | %17.07 | %0.01 | %0.00 | %0.01 |
| **ST060** | %24.82 | %0.00 | %0.00 | %0.00 |
| **ST061** | %21.78 | %0.00 | %0.00 | %0.00 |
| **ST062** | %12.05 | %0.00 | %0.00 | %0.00 |
| **ST063** | %6.91 | %0.00 | %0.00 | %0.00 |
| **ST064** | %19.64 | %0.00 | %0.00 | %0.00 |
| **ST065** | %17.83 | %0.00 | %0.00 | %0.00 |
| **ST066** | %25.73 | %0.01 | %0.00 | %0.01 |
| **ST067** | %17.38 | %0.14 | %0.00 | %0.14 |
| **ST068** | %27.48 | %0.00 | %0.00 | %0.00 |
| **ST069** | %21.84 | %0.00 | %0.00 | %0.00 |
| **ST070** | %26.35 | %0.00 | %0.00 | %0.00 |
| **ST071** | %14.05 | %0.00 | %0.00 | %0.00 |
| **ST072** | %10.90 | %0.00 | %0.00 | %0.00 |
| **ST073** | %16.03 | %0.00 | %0.00 | %0.00 |
| **ST074** | %17.38 | %0.00 | %0.00 | %0.00 |
| **ST075** | %14.13 | %0.00 | %0.00 | %0.00 |
| **ST076** | %3.80 | %0.00 | %0.00 | %0.00 |
| **ST077** | %13.27 | %0.00 | %0.00 | %0.00 |
| **ST078** | %14.95 | %0.00 | %0.00 | %0.00 |
| **ST079** | %9.96 | %0.00 | %0.00 | %0.00 |
| **ST080** | %17.28 | %0.01 | %0.00 | %0.01 |
| **ST081** | %5.57 | %0.00 | %0.00 | %0.00 |
| **ST082** | %30.23 | %0.00 | %0.00 | %0.00 |
| **ST083** | %34.47 | %0.00 | %0.00 | %0.00 |
| **ST084** | %22.54 | %0.00 | %0.00 | %0.00 |
| **ST085** | %26.58 | %0.00 | %0.00 | %0.00 |
| **ST086** | %11.92 | %0.00 | %0.00 | %0.00 |
| **ST087** | %26.18 | %0.00 | %0.00 | %0.00 |
| **ST088** | %23.76 | %0.00 | %0.00 | %0.00 |
| **ST089** | %2.61 | %0.00 | %0.00 | %0.00 |
| **ST090** | %19.13 | %0.00 | %0.00 | %0.00 |
| **ST091** | %22.23 | %0.00 | %0.00 | %0.00 |
| **ST092** | %22.68 | %0.00 | %0.00 | %0.00 |
| **ST093** | %23.42 | %0.00 | %0.00 | %0.00 |
| **ST094** | %10.65 | %0.00 | %0.00 | %0.00 |
| **ST095** | %21.29 | %0.00 | %0.00 | %0.00 |
| **ST096** | %26.04 | %0.00 | %0.00 | %0.00 |
| **ST097** | %22.26 | %0.00 | %0.00 | %0.00 |
| **ST098** | %0.21 | %0.00 | %0.00 | %0.00 |
| **ST099** | %34.75 | %0.00 | %0.00 | %0.00 |
| **ST100** | %17.64 | %0.00 | %0.00 | %0.00 |
| **ST101** | %9.27 | %0.00 | %0.00 | %0.00 |
| **ST102** | %7.50 | %0.00 | %0.00 | %0.00 |
| **ST103** | %14.36 | %0.01 | %0.00 | %0.01 |
| **ST104** | %6.16 | %0.00 | %0.00 | %0.00 |
| **ST105** | %25.64 | %0.27 | %0.00 | %0.27 |
| **ST106** | %17.29 | %0.00 | %0.00 | %0.00 |
| **ST107** | %45.53 | %0.00 | %0.00 | %0.00 |
| **ST108** | %24.67 | %0.00 | %0.00 | %0.00 |
| **ST109** | %46.95 | %0.06 | %0.00 | %0.05 |
| **ST110** | %24.74 | %0.00 | %0.00 | %0.00 |
| **ST111** | %16.41 | %0.00 | %0.00 | %0.00 |
| **ST112** | %14.90 | %0.01 | %0.00 | %0.01 |
| **ST113** | %10.36 | %0.00 | %0.00 | %0.00 |
| **ST114** | %16.92 | %0.00 | %0.00 | %0.00 |
| **ST115** | %21.67 | %0.00 | %0.00 | %0.00 |
| **ST116** | %19.12 | %0.00 | %0.00 | %0.00 |
| **ST117** | %15.89 | %0.00 | %0.00 | %0.00 |
| **ST118** | %18.54 | %0.00 | %0.00 | %0.00 |
| **ST119** | %9.04 | %0.00 | %0.00 | %0.00 |
| **ST120** | %25.94 | %0.00 | %0.00 | %0.00 |
| **ST121** | %26.10 | %0.38 | %0.00 | %0.38 |
| **ST122** | %25.69 | %0.04 | %0.00 | %0.04 |
| **ST123** | %15.80 | %0.00 | %0.00 | %0.00 |
| **ST124** | %21.64 | %0.00 | %0.00 | %0.00 |
| **ST125** | %32.00 | %0.00 | %0.00 | %0.00 |
| **ST126** | %164.63 | %52.84 | %16.65 | %43.42 |
| **ST127** | %23.39 | %0.00 | %0.00 | %0.00 |
| **ST128** | %12.85 | %0.43 | %0.01 | %0.41 |
| **ST129** | %24.75 | %0.00 | %0.00 | %0.00 |
| **ST130** | %20.07 | %0.00 | %0.00 | %0.00 |
| **ST131** | %10.33 | %0.00 | %0.00 | %0.00 |
| **ST132** | %35.57 | %0.07 | %0.00 | %0.07 |
| **ST133** | %24.00 | %0.00 | %0.00 | %0.00 |
| **ST134** | %7.02 | %0.00 | %0.00 | %0.00 |
| **ST135** | %58.44 | %1.41 | %0.07 | %1.34 |
| **ST136** | %16.69 | %0.00 | %0.00 | %0.00 |
| **ST137** | %19.34 | %0.00 | %0.00 | %0.00 |
| **ST138** | %21.78 | %0.00 | %0.00 | %0.00 |
| **ST139** | %20.49 | %0.00 | %0.00 | %0.00 |
| **ST140** | %22.19 | %0.00 | %0.00 | %0.00 |
| **ST141** | %22.76 | %0.00 | %0.00 | %0.00 |
| **ST142** | %5.29 | %0.00 | %0.00 | %0.00 |
| **ST143** | %27.47 | %0.00 | %0.00 | %0.00 |
| **ST144** | %28.08 | %0.00 | %0.00 | %0.00 |
| **ST145** | %4.41 | %0.00 | %0.00 | %0.00 |
| **ST146** | %20.63 | %0.00 | %0.00 | %0.00 |
| **ST147** | %19.19 | %0.00 | %0.00 | %0.00 |
| **ST148** | %16.91 | %0.00 | %0.00 | %0.00 |
| **ST149** | %17.83 | %0.00 | %0.00 | %0.00 |
| **ST150** | %15.12 | %0.01 | %0.00 | %0.01 |
| **ST151** | %31.32 | %0.00 | %0.00 | %0.00 |
| **ST152** | %11.51 | %0.00 | %0.00 | %0.00 |
| **ST153** | %34.66 | %0.00 | %0.00 | %0.00 |
| **ST154** | %14.69 | %0.00 | %0.00 | %0.00 |
| **ST155** | %6.15 | %0.00 | %0.00 | %0.00 |
| **ST156** | %5.24 | %0.00 | %0.00 | %0.00 |
| **ST157** | %28.23 | %0.00 | %0.00 | %0.00 |
| **ST158** | %13.41 | %0.01 | %0.00 | %0.01 |
| **ST159** | %36.19 | %4.70 | %0.37 | %4.34 |
| **ST160** | %75.99 | %26.36 | %5.13 | %22.38 |
| **ST161** | %53.53 | %0.46 | %0.02 | %0.44 |
| **ST162** | %32.07 | %0.00 | %0.00 | %0.00 |
| **ST163** | %28.46 | %0.00 | %0.00 | %0.00 |
| **ST164** | %60.60 | %8.25 | %0.18 | %8.08 |
| **ST165** | %23.28 | %0.00 | %0.00 | %0.00 |
| **ST166** | %29.70 | %0.00 | %0.00 | %0.00 |
| **ST167** | %17.69 | %0.01 | %0.00 | %0.01 |
| **ST168** | %31.09 | %0.00 | %0.00 | %0.00 |
| **ST169** | %31.08 | %0.00 | %0.00 | %0.00 |
| **ST170** | %24.20 | %0.00 | %0.00 | %0.00 |
| **ST171** | %10.34 | %0.00 | %0.00 | %0.00 |
| **ST172** | %26.08 | %0.00 | %0.00 | %0.00 |
| **ST173** | %34.97 | %0.00 | %0.00 | %0.00 |
| **ST174** | %26.23 | %0.00 | %0.00 | %0.00 |
| **ST175** | %35.52 | %0.00 | %0.00 | %0.00 |
| **ST176** | %12.17 | %0.01 | %0.00 | %0.01 |
| **ST177** | %21.36 | %0.00 | %0.00 | %0.00 |
| **ST178** | %23.86 | %0.00 | %0.00 | %0.00 |
| **ST179** | %44.59 | %0.00 | %0.00 | %0.00 |
| **ST180** | %35.75 | %0.01 | %0.00 | %0.01 |
| **ST181** | %18.36 | %0.00 | %0.00 | %0.00 |
| **ST182** | %25.20 | %0.00 | %0.00 | %0.00 |
| **ST183** | %23.38 | %0.00 | %0.00 | %0.00 |
| **ST184** | %29.88 | %0.00 | %0.00 | %0.00 |
| **ST185** | %20.06 | %0.00 | %0.00 | %0.00 |
| **ST186** | %17.79 | %0.00 | %0.00 | %0.00 |
| **ST187** | %26.69 | %0.00 | %0.00 | %0.00 |
| **ST188** | %29.01 | %0.00 | %0.00 | %0.00 |
| **ST189** | %53.50 | %0.07 | %0.00 | %0.07 |
| **ST190** | %20.31 | %0.00 | %0.00 | %0.00 |
| **ST191** | %21.41 | %0.00 | %0.00 | %0.00 |
| **ST192** | %31.87 | %0.00 | %0.00 | %0.00 |
| **ST193** | %26.11 | %0.00 | %0.00 | %0.00 |
| **ST194** | %13.77 | %0.00 | %0.00 | %0.00 |
| **ST195** | %35.31 | %0.00 | %0.00 | %0.00 |
| **ST196** | %25.80 | %0.00 | %0.00 | %0.00 |
| **ST197** | %26.29 | %0.00 | %0.00 | %0.00 |
| **ST198** | %26.29 | %0.00 | %0.00 | %0.00 |
| **ST199** | %28.88 | %0.00 | %0.00 | %0.00 |
| **ST200** | %30.56 | %0.00 | %0.00 | %0.00 |
| **ST201** | %34.73 | %0.00 | %0.00 | %0.00 |
| **ST202** | %9.41 | %0.00 | %0.00 | %0.00 |
| **ST203** | %33.25 | %0.00 | %0.00 | %0.00 |
| **ST204** | %35.47 | %0.00 | %0.00 | %0.00 |
| **ST205** | %34.34 | %0.00 | %0.00 | %0.00 |
| **ST206** | %34.36 | %0.00 | %0.00 | %0.00 |
| **ST207** | %18.57 | %0.30 | %0.01 | %0.29 |
| **ST208** | %18.51 | %0.09 | %0.00 | %0.09 |
| **ST209** | %30.16 | %0.00 | %0.00 | %0.00 |
| **ST210** | %21.60 | %0.00 | %0.00 | %0.00 |
| **ST211** | %34.38 | %0.00 | %0.00 | %0.00 |
| **ST212** | %26.20 | %0.00 | %0.00 | %0.00 |
| **ST213** | %22.09 | %0.95 | %0.04 | %0.90 |
| **ST214** | %29.70 | %0.00 | %0.00 | %0.00 |
| **ST215** | %24.21 | %0.27 | %0.01 | %0.27 |
| **ST216** | %45.59 | %0.00 | %0.00 | %0.00 |
| **ST217** | %18.33 | %2.13 | %0.01 | %2.12 |
| **ST218** | %36.14 | %0.00 | %0.00 | %0.00 |
| **ST219** | %39.86 | %0.00 | %0.00 | %0.00 |
| **ST220** | %42.61 | %0.00 | %0.00 | %0.00 |
| **ST221** | %46.99 | %0.00 | %0.00 | %0.00 |
| **ST222** | %43.82 | %0.00 | %0.00 | %0.00 |
| **ST223** | %31.15 | %0.00 | %0.00 | %0.00 |
| **ST224** | %33.79 | %0.00 | %0.00 | %0.00 |
| **ST225** | %46.69 | %0.00 | %0.00 | %0.00 |
| **ST226** | %27.65 | %0.00 | %0.00 | %0.00 |
| **ST227** | %44.47 | %0.00 | %0.00 | %0.00 |
| **ST228** | %28.06 | %0.00 | %0.00 | %0.00 |
| **ST229** | %35.87 | %0.00 | %0.00 | %0.00 |
| **ST230** | %31.67 | %0.00 | %0.00 | %0.00 |
| **ST231** | %28.26 | %0.00 | %0.00 | %0.00 |
| **ST232** | %45.73 | %0.00 | %0.00 | %0.00 |
| **ST233** | %41.34 | %0.00 | %0.00 | %0.00 |
| **ST234** | %50.00 | %0.00 | %0.00 | %0.00 |
| **ST235** | %39.57 | %0.00 | %0.00 | %0.00 |
| **ST236** | %43.04 | %0.00 | %0.00 | %0.00 |
| **ST237** | %56.00 | %0.00 | %0.00 | %0.00 |
| **ST238** | %29.36 | %0.00 | %0.00 | %0.00 |
| **ST239** | %47.73 | %0.00 | %0.00 | %0.00 |
| **ST240** | %26.89 | %0.00 | %0.00 | %0.00 |
| **ST241** | %0.07 | %0.00 | %0.00 | %0.00 |
| **ST242** | %40.78 | %0.00 | %0.00 | %0.00 |
| **ST243** | %29.21 | %0.00 | %0.00 | %0.00 |
| **ST244** | %44.07 | %0.00 | %0.00 | %0.00 |
| **ST245** | %45.24 | %0.00 | %0.00 | %0.00 |
| **ST246** | %34.35 | %0.00 | %0.00 | %0.00 |
| **ST247** | %30.90 | %0.00 | %0.00 | %0.00 |
| **ST248** | %39.78 | %0.00 | %0.00 | %0.00 |
| **ST249** | %24.45 | %0.00 | %0.00 | %0.00 |
| **ST250** | %25.78 | %0.00 | %0.00 | %0.00 |
| **ST251** | %38.01 | %0.00 | %0.00 | %0.00 |
| **ST252** | %46.66 | %0.00 | %0.00 | %0.00 |
| **ST253** | %45.08 | %0.00 | %0.00 | %0.00 |
| **ST254** | %26.09 | %0.00 | %0.00 | %0.00 |
| **ST255** | %43.47 | %0.00 | %0.00 | %0.00 |
| **ST256** | %57.75 | %0.00 | %0.00 | %0.00 |
| **ST257** | %38.83 | %0.00 | %0.00 | %0.00 |
| **ST258** | %31.00 | %0.02 | %0.00 | %0.02 |
| **ST259** | %33.16 | %0.00 | %0.00 | %0.00 |
| **ST260** | %40.77 | %0.00 | %0.00 | %0.00 |
| **ST261** | %34.75 | %0.00 | %0.00 | %0.00 |
| **ST262** | %41.40 | %0.00 | %0.00 | %0.00 |
| **ST263** | %41.19 | %0.00 | %0.00 | %0.00 |
| **ST264** | %37.25 | %0.00 | %0.00 | %0.00 |
| **ST265** | %49.21 | %0.00 | %0.00 | %0.00 |
| **ST266** | %35.31 | %0.00 | %0.00 | %0.00 |
| **ST267** | %44.63 | %0.00 | %0.00 | %0.00 |
| **ST268** | %35.94 | %0.00 | %0.00 | %0.00 |
| **ST269** | %34.01 | %0.00 | %0.00 | %0.00 |
| **ST270** | %29.44 | %0.01 | %0.00 | %0.01 |
| **ST271** | %33.30 | %0.00 | %0.00 | %0.00 |
| **ST272** | %77.24 | %0.01 | %0.00 | %0.01 |
| **ST273** | %21.86 | %0.00 | %0.00 | %0.00 |
| **ST274** | %43.67 | %0.00 | %0.00 | %0.00 |
| **ST275** | %39.67 | %0.00 | %0.00 | %0.00 |
| **ST276** | %32.81 | %0.00 | %0.00 | %0.00 |
| **ST277** | %35.61 | %0.10 | %0.00 | %0.10 |
| **ST278** | %29.69 | %0.00 | %0.00 | %0.00 |
| **ST279** | %30.07 | %0.00 | %0.00 | %0.00 |
| **ST280** | %20.93 | %0.00 | %0.00 | %0.00 |
| **ST281** | %19.66 | %0.00 | %0.00 | %0.00 |
| **ST282** | %40.33 | %0.00 | %0.00 | %0.00 |
| **ST283** | %7.51 | %0.00 | %0.00 | %0.00 |
| **ST284** | %44.66 | %0.00 | %0.00 | %0.00 |
| **ST285** | %49.36 | %0.00 | %0.00 | %0.00 |
| **ST286** | %43.03 | %0.00 | %0.00 | %0.00 |
| **ST287** | %31.74 | %0.00 | %0.00 | %0.00 |
| **ST288** | %64.40 | %0.00 | %0.00 | %0.00 |
| **ST289** | %50.62 | %0.00 | %0.00 | %0.00 |
| **ST290** | %50.06 | %0.00 | %0.00 | %0.00 |
| **ST291** | %45.21 | %0.00 | %0.00 | %0.00 |
| **ST292** | %40.06 | %0.00 | %0.00 | %0.00 |
| **ST293** | %37.79 | %0.00 | %0.00 | %0.00 |
| **ST294** | %25.96 | %0.00 | %0.00 | %0.00 |
| **ST295** | %89.05 | %2.14 | %0.03 | %2.11 |
| **ST296** | %45.74 | %0.00 | %0.00 | %0.00 |
| **ST297** | %4.38 | %0.00 | %0.00 | %0.00 |
| **ST298** | %44.68 | %0.00 | %0.00 | %0.00 |
| **ST299** | %40.16 | %0.00 | %0.00 | %0.00 |
| **ST300** | %39.22 | %0.00 | %0.00 | %0.00 |
| **ST301** | %21.44 | %0.00 | %0.00 | %0.00 |
| **ST302** | %39.22 | %0.00 | %0.00 | %0.00 |
| **ST303** | %44.47 | %0.00 | %0.00 | %0.00 |
| **ST304** | %43.40 | %0.00 | %0.00 | %0.00 |
| **ST305** | %85.81 | %0.00 | %0.00 | %0.00 |
| **ST306** | %51.01 | %0.00 | %0.00 | %0.00 |

**Appendix C**

**Table 17.** Random Forest Model Results

| **Company Name** | **Non-Default Probability** | **Default Probability** | **Situation** | **Company Name** | **Non-Default Probability** | **Default Probability** | **Situation** |
| --- | --- | --- | --- | --- | --- | --- | --- |
| **ST101** | %41 | %59 | Default | **ST257** | %99 | %1 | Non-Default |
| **ST102** | %26 | %74 | Default | **ST258** | %83 | %17 | Non-Default |
| **ST005** | %33 | %67 | Default | **ST259** | %93 | %7 | Non-Default |
| **ST059** | %73 | %27 | Non-Default | **ST260** | %99 | %1 | Non-Default |
| **ST211** | %91 | %9 | Non-Default | **ST261** | %98 | %2 | Non-Default |
| **ST212** | %100 | %0 | Non-Default | **ST262** | %90 | %10 | Non-Default |
| **ST213** | %85 | %15 | Non-Default | **ST263** | %98 | %2 | Non-Default |
| **ST214** | %98 | %2 | Non-Default | **ST264** | %98 | %2 | Non-Default |
| **ST215** | %81 | %19 | Non-Default | **ST265** | %69 | %31 | Non-Default |
| **ST216** | %60 | %40 | Non-Default | **ST266** | %99 | %1 | Non-Default |
| **ST217** | %73 | %27 | Non-Default | **ST267** | %96 | %4 | Non-Default |
| **ST218** | %99 | %1 | Non-Default | **ST268** | %96 | %4 | Non-Default |
| **ST219** | %94 | %6 | Non-Default | **ST269** | %96 | %4 | Non-Default |
| **ST220** | %96 | %4 | Non-Default | **ST270** | %76 | %24 | Non-Default |
| **ST221** | %92 | %8 | Non-Default | **ST271** | %99 | %1 | Non-Default |
| **ST222** | %93 | %7 | Non-Default | **ST272** | %97 | %3 | Non-Default |
| **ST223** | %97 | %3 | Non-Default | **ST273** | %98 | %2 | Non-Default |
| **ST224** | %99 | %1 | Non-Default | **ST274** | %95 | %5 | Non-Default |
| **ST225** | %72 | %28 | Non-Default | **ST275** | %100 | %0 | Non-Default |
| **ST226** | %100 | %0 | Non-Default | **ST276** | %59 | %41 | Non-Default |
| **ST227** | %87 | %13 | Non-Default | **ST277** | %97 | %3 | Non-Default |
| **ST228** | %86 | %14 | Non-Default | **ST278** | %99 | %1 | Non-Default |
| **ST229** | %94 | %6 | Non-Default | **ST279** | %73 | %27 | Non-Default |
| **ST230** | %71 | %29 | Non-Default | **ST280** | %54 | %46 | Non-Default |
| **ST231** | %84 | %16 | Non-Default | **ST281** | %99 | %1 | Non-Default |
| **ST232** | %92 | %8 | Non-Default | **ST282** | %94 | %6 | Non-Default |
| **ST233** | %85 | %15 | Non-Default | **ST283** | %63 | %37 | Non-Default |
| **ST234** | %86 | %14 | Non-Default | **ST284** | %88 | %12 | Non-Default |
| **ST235** | %91 | %9 | Non-Default | **ST285** | %67 | %33 | Non-Default |
| **ST236** | %85 | %15 | Non-Default | **ST286** | %99 | %1 | Non-Default |
| **ST237** | %72 | %28 | Non-Default | **ST287** | %66 | %34 | Non-Default |
| **ST238** | %32 | %68 | Default | **ST288** | %46 | %54 | Default |
| **ST239** | %46 | %54 | Default | **ST289** | %69 | %31 | Non-Default |
| **ST240** | %52 | %48 | Non-Default | **ST290** | %100 | %0 | Non-Default |
| **ST241** | %35 | %65 | Default | **ST291** | %68 | %32 | Non-Default |
| **ST242** | %35 | %65 | Default | **ST292** | %97 | %3 | Non-Default |
| **ST243** | %36 | %64 | Default | **ST293** | %98 | %2 | Non-Default |
| **ST244** | %82 | %18 | Non-Default | **ST294** | %74 | %26 | Non-Default |
| **ST245** | %97 | %3 | Non-Default | **ST295** | %75 | %25 | Non-Default |
| **ST246** | %87 | %13 | Non-Default | **ST296** | %95 | %5 | Non-Default |
| **ST247** | %99 | %1 | Non-Default | **ST297** | %99 | %1 | Non-Default |
| **ST248** | %90 | %10 | Non-Default | **ST298** | %65 | %35 | Non-Default |
| **ST249** | %98 | %2 | Non-Default | **ST299** | %100 | %0 | Non-Default |
| **ST250** | %86 | %14 | Non-Default | **ST300** | %90 | %10 | Non-Default |
| **ST251** | %99 | %1 | Non-Default | **ST301** | %93 | %7 | Non-Default |
| **ST252** | %97 | %3 | Non-Default | **ST302** | %100 | %0 | Non-Default |
| **ST253** | %100 | %0 | Non-Default | **ST303** | %98 | %2 | Non-Default |
| **ST254** | %100 | %0 | Non-Default | **ST304** | %98 | %2 | Non-Default |
| **ST255** | %94 | %6 | Non-Default | **ST305** | %98 | %2 | Non-Default |
| **ST256** | %100 | %0 | Non-Default | **ST306** | %65 | %35 | Non-Default |

**Appendix D**

**Table 18.** Gradient Boosted Decision Tree Model Results

| **Company Name** | **Non-Default Probability** | **Default Probability** | **Situation** | **Company Name** | **Non-Default Probability** | **Default Probability** | **Situation** |
| --- | --- | --- | --- | --- | --- | --- | --- |
| **ST101** | %7.46 | %92.54 | Default | **ST257** | %99.70 | %0.30 | Non-Default |
| **ST102** | %12.25 | %87.75 | Default | **ST258** | %99.63 | %0.37 | Non-Default |
| **ST005** | %2.08 | %97.92 | Default | **ST259** | %99.64 | %0.36 | Non-Default |
| **ST059** | %84.45 | %15.55 | Non-Default | **ST260** | %99.78 | %0.22 | Non-Default |
| **ST211** | %99.60 | %0.40 | Non-Default | **ST261** | %99.40 | %0.60 | Non-Default |
| **ST212** | %99.70 | %0.30 | Non-Default | **ST262** | %99.36 | %0.64 | Non-Default |
| **ST213** | %95.28 | %4.72 | Non-Default | **ST263** | %99.70 | %0.30 | Non-Default |
| **ST214** | %99.75 | %0.25 | Non-Default | **ST264** | %99.63 | %0.37 | Non-Default |
| **ST215** | %93.89 | %6.11 | Non-Default | **ST265** | %7.55 | %92.45 | Default |
| **ST216** | %8.93 | %91.07 | Default | **ST266** | %99.78 | %0.22 | Non-Default |
| **ST217** | %96.46 | %3.54 | Non-Default | **ST267** | %99.75 | %0.25 | Non-Default |
| **ST218** | %99.70 | %0.30 | Non-Default | **ST268** | %99.68 | %0.32 | Non-Default |
| **ST219** | %99.75 | %0.25 | Non-Default | **ST269** | %99.70 | %0.30 | Non-Default |
| **ST220** | %99.56 | %0.44 | Non-Default | **ST270** | %81.63 | %18.37 | Non-Default |
| **ST221** | %99.21 | %0.79 | Non-Default | **ST271** | %99.75 | %0.25 | Non-Default |
| **ST222** | %99.56 | %0.44 | Non-Default | **ST272** | %99.70 | %0.30 | Non-Default |
| **ST223** | %99.28 | %0.72 | Non-Default | **ST273** | %99.75 | %0.25 | Non-Default |
| **ST224** | %99.75 | %0.25 | Non-Default | **ST274** | %99.70 | %0.30 | Non-Default |
| **ST225** | %99.06 | %0.94 | Non-Default | **ST275** | %99.74 | %0.26 | Non-Default |
| **ST226** | %99.75 | %0.25 | Non-Default | **ST276** | %74.70 | %25.30 | Non-Default |
| **ST227** | %99.53 | %0.47 | Non-Default | **ST277** | %99.70 | %0.30 | Non-Default |
| **ST228** | %98.21 | %1.79 | Non-Default | **ST278** | %99.74 | %0.26 | Non-Default |
| **ST229** | %99.70 | %0.30 | Non-Default | **ST279** | %99.37 | %0.63 | Non-Default |
| **ST230** | %8.59 | %91.41 | Default | **ST280** | %63.13 | %36.87 | Non-Default |
| **ST231** | %99.73 | %0.27 | Non-Default | **ST281** | %99.75 | %0.25 | Non-Default |
| **ST232** | %99.56 | %0.44 | Non-Default | **ST282** | %99.62 | %0.38 | Non-Default |
| **ST233** | %95.78 | %4.22 | Non-Default | **ST283** | %91.72 | %8.28 | Non-Default |
| **ST234** | %99.56 | %0.44 | Non-Default | **ST284** | %99.70 | %0.30 | Non-Default |
| **ST235** | %98.75 | %1.25 | Non-Default | **ST285** | %5.95 | %94.05 | Default |
| **ST236** | %99.63 | %0.37 | Non-Default | **ST286** | %99.49 | %0.51 | Non-Default |
| **ST237** | %8.51 | %91.49 | Default | **ST287** | %7.55 | %92.45 | Default |
| **ST238** | %31.29 | %68.71 | Default | **ST288** | %98.17 | %1.83 | Non-Default |
| **ST239** | %10.42 | %89.58 | Default | **ST289** | %13.85 | %86.15 | Default |
| **ST240** | %79.80 | %20.20 | Non-Default | **ST290** | %99.78 | %0.22 | Non-Default |
| **ST241** | %5.29 | %94.71 | Default | **ST291** | %8.59 | %91.41 | Default |
| **ST242** | %10.62 | %89.38 | Default | **ST292** | %99.61 | %0.39 | Non-Default |
| **ST243** | %7.69 | %92.31 | Default | **ST293** | %99.64 | %0.36 | Non-Default |
| **ST244** | %96.82 | %3.18 | Non-Default | **ST294** | %97.42 | %2.25 | Non-Default |
| **ST245** | %99.60 | %0.40 | Non-Default | **ST295** | %98.36 | %1.64 | Non-Default |
| **ST246** | %99.56 | %0.44 | Non-Default | **ST296** | %99.75 | %0.25 | Non-Default |
| **ST247** | %99.70 | %0.30 | Non-Default | **ST297** | %99.11 | %0.89 | Non-Default |
| **ST248** | %99.71 | %0.29 | Non-Default | **ST298** | %4.55 | %95.45 | Default |
| **ST249** | %99.64 | %0.36 | Non-Default | **ST299** | %99.75 | %0.25 | Non-Default |
| **ST250** | %97.91 | %2.09 | Non-Default | **ST300** | %99.22 | %0.78 | Non-Default |
| **ST251** | %99.78 | %0.22 | Non-Default | **ST301** | %99.70 | %0.30 | Non-Default |
| **ST252** | %99.70 | %0.30 | Non-Default | **ST302** | %99.70 | %0.30 | Non-Default |
| **ST253** | %99.75 | %0.25 | Non-Default | **ST303** | %99.75 | %0.25 | Non-Default |
| **ST254** | %99.68 | %0.32 | Non-Default | **ST304** | %99.68 | %0.32 | Non-Default |
| **ST255** | %99.74 | %0.26 | Non-Default | **ST305** | %99.78 | %0.22 | Non-Default |
| **ST256** | %99.75 | %0.25 | Non-Default | **ST306** | %6.32 | %93.68 | Default |
